# The Pagerank-Index: Going beyond Citation Counts in Quantifying Scientific Impact of Researchers

**DOI:** 10.1371/journal.pone.0134794

**Published:** 2015-08-19

**Authors:** Upul Senanayake, Mahendra Piraveenan, Albert Zomaya

**Affiliations:** 1 Centre for Complex Systems Research, Faculty of Engineering and IT, The University of Sydney, NSW 2006, Australia; 2 Centre for Complex Systems Research, Faculty of Engineering and IT, The University of Sydney, NSW 2006, Australia; 3 Centre for Distributed and High Performance Computing, School of Information Technologies, The University of Sydney, NSW 2006, Australia; VU University Amsterdam, NETHERLANDS

## Abstract

Quantifying and comparing the scientific output of researchers has become critical for governments, funding agencies and universities. Comparison by reputation and direct assessment of contributions to the field is no longer possible, as the number of scientists increases and traditional definitions about scientific fields become blurred. The h-index is often used for comparing scientists, but has several well-documented shortcomings. In this paper, we introduce a new index for measuring and comparing the publication records of scientists: the pagerank-index (symbolised as *π*). The index uses a version of pagerank algorithm and the citation networks of papers in its computation, and is fundamentally different from the existing variants of h-index because it considers not only the number of citations but also the actual impact of each citation. We adapt two approaches to demonstrate the utility of the new index. Firstly, we use a simulation model of a community of authors, whereby we create various ‘groups’ of authors which are different from each other in inherent publication habits, to show that the pagerank-index is fairer than the existing indices in three distinct scenarios: (i) when authors try to ‘massage’ their index by publishing papers in low-quality outlets primarily to self-cite other papers (ii) when authors collaborate in large groups in order to obtain more authorships (iii) when authors spend most of their time in producing genuine but low quality publications that would massage their index. Secondly, we undertake two real world case studies: (i) the evolving author community of quantum game theory, as defined by Google Scholar (ii) a snapshot of the high energy physics (HEP) theory research community in arXiv. In both case studies, we find that the list of top authors vary very significantly when h-index and pagerank-index are used for comparison. We show that in both cases, authors who have collaborated in large groups and/or published less impactful papers tend to be comparatively favoured by the h-index, whereas the pagerank-index highlights authors who have made a relatively small number of definitive contributions, or written papers which served to highlight the link between diverse disciplines, or typically worked in smaller groups. Thus, we argue that the pagerank-index is an inherently fairer and more nuanced metric to quantify the publication records of scientists compared to existing measures.

## Introduction

Comparing the research output of scientists and academics could be termed a ‘necessary evil’ in the world scientific community today. Indeed, scientists are supposed to work for the betterment of humanity and how well they succeed in their endeavour cannot easily be measured. Yet, it is an accepted norm to publish scientific research via peer-reviewed papers and other citable documents, in addition to producing tangible goods, services, or results which may lead to the emancipation of mankind. Government funded research councils, corporate entities, universities and other research organisations primarily rely on the publication records of scientists to make funding and employment decisions. These entities use well known metrics to compare the scientific output of researchers. Besides, such metrics are now accepted as indicators of the prestige and standing of a researcher in the scientific community. Popular scientific databases such as Google Scholar or ISI Web of Science compute and display these metrics, so that interested parties may understand the apparent standing of a researcher by looking them up.

Some examples of metrics used to evaluate the publication record of a scientist are the number of publications, total number of citations, the number of citations per paper, the i10-index [1] and the h-index. The h-index is perhaps the most sophisticated and nuanced measure among these, since it accounts for both the quality and the quantity of a scientists’ research publications. The h-index was defined by Hirsch [2] as the number of papers with ≥ *h* number of citations. There are evident drawbacks in the other measures mentioned above which the h-index has addressed. For instance, quality or the impact of the papers is not taken in to account if number of papers is used while a few papers with a high number of citations co-authored by many authors can inflate the number of citations measure. Low productivity is rewarded by the number of citations per paper measure. Hirsch’s h-index became successful because he pointed out that the h-index addresses these drawbacks by considering both the productivity (number of papers) and impact (citations a paper receives) of the scientist concerned.

Since its introduction, h-index has been widely accepted by the scientific community [3], and some evidence suggests that the scientists who are successful in grants and employability tend to have a comparatively high h-index [4]. However, many shortcomings of h-index have been pointed out creating considerable debate over the use of h-index [5–7], and many variants of h-index have been proposed to address these. For example, h-index depends on the time span of the career of a scientist and hence a brilliant scientist whose career is at the early stages can be reflected unfavorably [2]. Hirsch suggested a time compensated h-index to address this issue, though in practise this is not much used. Egghe [6], in his work an year later, contended that once a publication belongs in the h-core (among the papers ranked h or higher), the h-index becomes indifferent to any further citations to that paper. This penalizes the scientists who have high-impact papers and is evidenced by the difference between the lower bound of citations, *h* ^2^, proposed by the h-index, and the actual number of citations. Therefore Egghe suggested the use of g-index which is defined as the highest number *g* of papers that together received *g* ^2^ or more citations [6]. Similarly, the R-index and AR index introduced by Jin et al. [8] measure the citation intensity of the h-core, while also considering the age of the papers. Jin et al. suggested that these measures could be used together with the h-index to complement each other. There are other variants which address similar issues, as summarized by Van Rann [9] and Waltman [10]. A review of the existing variants of h-index by Bornmann et al. [3] suggests that all these variants fall into two types: those that describe the size of the h-core, and those that describe the impact of the h-core. The first type redefines the size of the h-core and typically makes it larger (such as g-index), or adds additional information about it (such as the R-index) while the second type analyses the citation count of the h-core, negating the disadvantage attributed to scientists who have a few high-impact papers.

However, a fundamental issue not addressed by all these metrics is that they still treat all citations equally. Yet, it is perfectly clear that a citation by a paper from a highly regarded journal, such as *Nature*, should be treated differently from a citation by a workshop paper or a technical report. If this does not happen, locally famous authors whose research does not have global impact but gets cited by their colleagues in their country or research circle can get rewarded. This is likely to happen, for example, in developing countries with relatively fewer universities and large but locally tight-knit research communities. Moreover, if all citations are treated equally, then ‘massaging’ the h-index becomes possible by publishing papers, in whatever available forum, purely with the intention of citing other papers by the same group of authors or collaborators (by ‘scratching each others’ back’, as it were). This process has the danger of encouraging the creation of a huge body of academic papers nobody reads, let alone utilizes, for further research or application.

**Fig 1:**
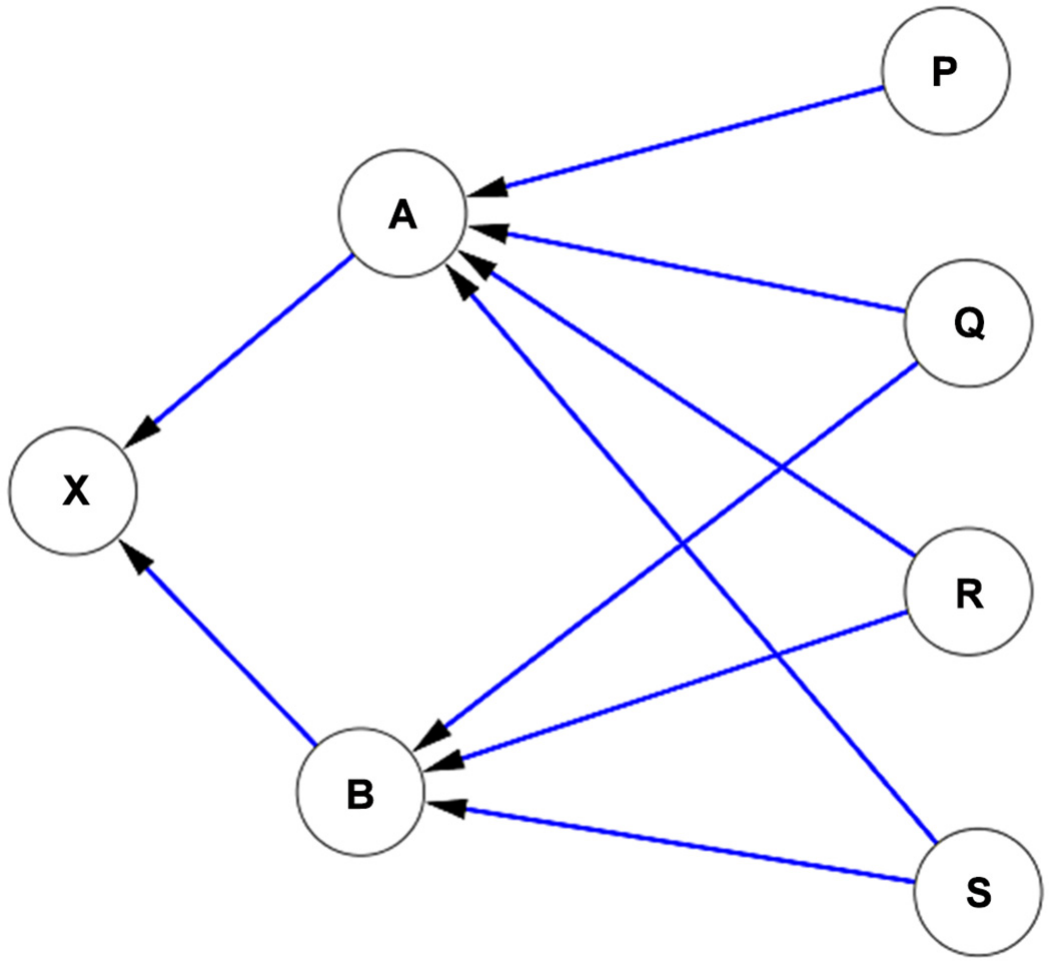
A citation network of documents with differing impacts. Document ‘A’ is a document with seemingly high impact: therefore citation from document A to document X could be weighed by the ‘impact’ of document A. However, it is clear that document A receives its high impact status from documents P, Q, R and S, which are themselves low impact documents. The documents P, Q, R and S could have been deliberately created to give more credibility to A. Similarly, document B which is also a high impact document receives its high impact status from low impact documents. Therefore, the citation counts of documents cannot be directly used to weigh the citations, since these weights themselves could be manipulated. A more nuanced approach is therefore necessary.

At first sight it may appear that this issue could be addressed simply by giving weights to citations in a straightforward manner, for e.g., by using journal impact factors. However, this approach has many pitfalls. There is considerable debate about the dependability of the impact factors of journals [11], and we cannot therefore consider that impact factors are sacrosanct. In any case, conference papers and relatively new journals do not have impact factors, and conference ranking systems are even less reliable, and using these essentially becomes an exercise in politics. There is no guarantee either, that a paper which is published in a journal with high impact factor will itself have high impact. Even if the number of citations received by the citing document is used to weigh the citation in a dynamic manner, it is possible that these weights could be manipulated again by citing the ‘citing document’ with less impactful documents: thus the influence of such documents only becomes indirect and secondary, and does not altogether diminish. This is portrayed in Fig 1. It is imperative, therefore, to introduce an index which is inherently above manipulation, has infinite levels of feedback to avoid secondary or tertiary manipulation, and where the ‘real’ impact of the citing document (not the nominal and generic impact factor of the journal in which it is published) is factored in. This could only be done by fully utilising the overall (dynamic) citation network, and by using an elegant algorithm which has had proven success in measuring the impact of single nodes in networks with infinite feedback loops.

In this paper, therefore, we introduce the pagerank-index (denoted as *π*), which is designed to address the drawbacks of existing indices by utilizing the underlying citation network dynamics. The index is calculated by running the well-known pagerank algorithm [12] on the evolving citation network to give scores to papers. Our goal is to formulate and test a fairer metric which rewards true excellence by giving higher weight to citations from documents with higher academic standing. We use the original form of pagerank algorithm to come up with pagerank values for each publication(Indeed, there have been a few isolated attempts to use pagerank algorithm or its variants to find high-impact papers in individual fields, such as [13–15]. However, justification of such use was limited to pointing out the reputation of the papers were thus uncovered, and in any case these studies did not propose or use an author ranking index. In this paper, by contrast, we propose a metric which compares researchers and provide a generic and principled study to justify it.), and the score of an author is calculated as a weighted sum of the scores of the papers he/she has written. The pagerank-index is expressed as a percentile, because unlike the h-index, the raw values computed by this measure may not make intuitive sense. On the other hand, the percentile form paves the way for easier understanding of the standing of scientists at a glance, and could be computed for the overall scientific community or any subset thereof, in terms of research field, country etc. Thus it is a generic tool.

The commercial success of Google as a search engine is in large part attributed to the pagerank algorithm [12] used within it. The premise behind pagerank algorithm is that it uses the number of links pointing to a web page, as well as the relative standing of pages from where the links originate, to determine the rank of a web page to be displayed in a Google search. In Google search engine, once a user enters a keyword or phrase, all websites which have that keyword are listed, and pagerank is used to rank these websites. Those pages which are linked from other pages with high ‘pagerank’ are awarded high scores, while the score is diluted if the link is from a page which links to too many other pages. Importantly, a web page designer cannot increase the pagerank of her page by providing links to it from many empty or purpose-less websites, which exist only to boost the pagerank of other pages. Therefore, a version of pagerank algorithm is suited for analysing scientific citation networks as well, and in this paper we use the publicly available version, which is generally held to have been proposed first by Page and Brin [12], though it could be observed that a similar algorithm is contained in the earlier work of Pinksi and Narin [16].

We undertake a detailed analysis to demonstrate the utility of the pagerank-index. On one hand, we develop and employ a realistic simulation system which synthesizes the evolution process of citation and collaboration networks in an emerging field. We demonstrate that the pagerank-index is fairer than h-index in many scenarios, which we simulate, in which the authors may try to gain unfair advantage by massaging or manipulating their h-index. In particular, we show that while it is possible to massage h-index by publishing papers in low-impact forums which are used simply to cite other papers of the same group of authors, this is largely impossible with pagerank-index. We also show that while h-index rewards collaboration and authors who focus on quantity, the pagerank-index is much more balanced and equally rewards individual brilliance and quality of papers. On the other hand, we demonstrate the utility of the new index by applying it to two real-world data sets. The first one is an evolving citation network specific to Quantum Game Theory field, defined according to the dedicated Google scholar profile, which we have curated from Google scholar. This network gives us valuable insights about pagerank-index because we can examine the evolution of the pagerank-index against time. We also examined the high energy physics theory dataset from arXiv (will be referred as HEP-TH dataset from here onwards) which has 29555 papers and 15332 authors covering the papers in the period from January 1993 to April 2003 [17]. Analysing these two datasets, we observe that the pagerank-index mitigates a number of scenarios where brilliant scientists are, arguably unfairly, given lower h-index scores. For example, we show that the h-index is heavily influenced by the number of authors of a paper, so that scientists could benefit by working in larger groups, whereby the pagerank-index neutralises this effect and highlights the contributions of smaller groups of authors. Similarly, authors who wrote papers which perhaps received relatively smaller number of citations, and acted as ‘bridges’ to hitherto unrelated fields, were highlighted by using pagerank-index. Further, as mentioned before, authors who received smaller number of citations, but mainly from papers which were highly cited themselves, were highlighted by using pagerank-index. Therefore, we demonstrate that the pagerank-index is a much ‘fairer’ metric for ranking scientists, and captures a broader interpretation of meaningful contribution to the respective field than simply cultivating citations from whatever source.

The rest of the paper is organised as follows: in the *Methods* section, we describe how the pagerank-index is defined and could be computed, and provide detailed justifications for our definitions. In the *Analysis* section, first we describe in detail the simulation experiments we conducted to model author behaviour, and the results we obtained, and how these results justify the new measure. We then describe the quantum game theory dataset, and how high-performing scientists could be better identified in this evolving field by using the pagerank-index. In the next subsection, we use the larger HEP-TH dataset similarly to provide further justification for the new index. In the *Discussion and Conclusions* section, we offer further discussion about our results, and present our overall conclusions.

## Methods

**Fig 2:**
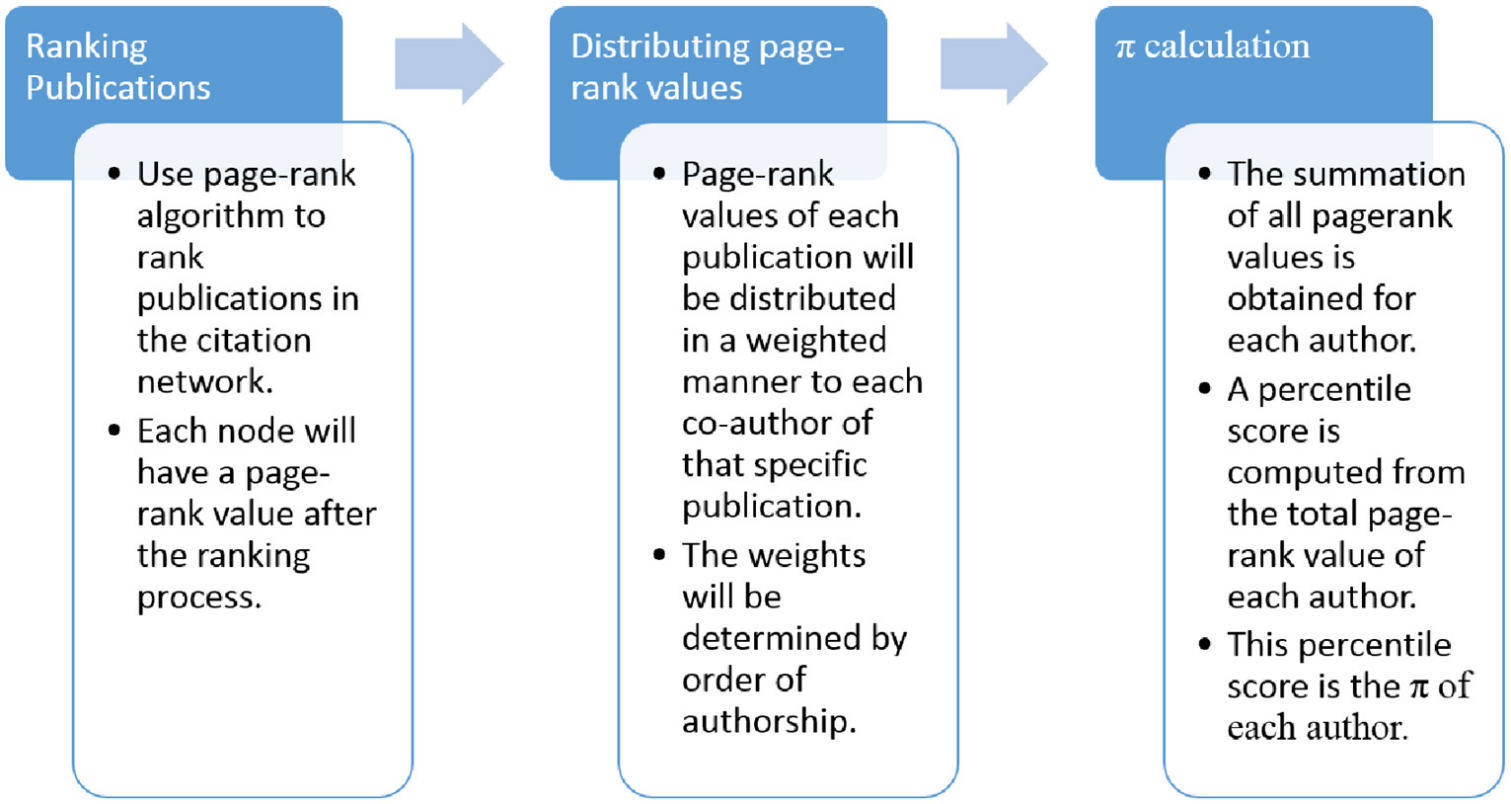
The process of computing pagerank-index. Stage I involves running pagerank algorithm on the citation network and obtaining a pagerank value for each paper. Stage II involves assigning a suitably weighted proportion of each pagerank value to the authors of the corresponding paper. Stage III involves summing all pagerank value ‘shares’ an author has obtained, comparing it with other authors in the community and assigning a percentile value to that author accordingly. The considered community could be the world scientific community at large, or a subset thereof.

In this section we define the pagerank-index and explain its computation. The pagerank-index *π* can be defined as the individual percentile ranking of a scientist in a scholarly database based on their cumulative weighted contribution from each publication they have co-authored, whereby publications are ranked using page-rank algorithm and the individual contribution from a publication is measured as the weighted proportion of the page-rank value of that publication. This can be further illustrated as a process by Fig 2. The process has three stages: (i) computing the page-rank value of each paper in the system (each node in the citation network) (ii) assigning weighted proportions of such values to each author in the system (each node in the collaboration network) (iii) computing the author pagerank-index as a percentile.

In the first stage, publications are ranked using the page-rank algorithm as described by Larry Page and Sergei Brin [12] which can be interpreted to mimic the behavior of a random surfer in the world wide web. The same interpretation holds for ‘citing behavior’ of scientists as well. We use page-rank algorithm as described by Eq (1) where $P_{i}^{t}$ is the pagerank of node *i* at time *t*, *A*_*ij*_ is the adjacency matrix of the network, *k*_out_(*j*) is the number of outgoing links from node *j* and *α* is a reset parameter. *N* is the number of nodes in the network.

$$\begin{array}{c} P_{i}^{t}=\frac{1-\alpha }{N}+\alpha \sum_{j}\frac{A_{ij}P_{j}^{\left(t-1\right)}}{k_{out}\left(j\right)} \end{array}$$ (1)

As has been elaborated, page-rank algorithm essentially measures the weight carried by each citation (reference) in the citation network, divided by the number of citations made by the citing document, and returns a decimal number to each document (node) in the citation network which we identify as the page-rank value of each node. To compute the page-rank value of each node (paper), we executed Page-rank algorithm multiple times until it reached steady state at time *t* = *τ* by satisfying the condition in Eq (2). We decided the margin-of-error *ϵ* to be 0.0001 which gives us the required consistency over multiple executions of the page-rank algorithm. After this condition is met, we use the stable-state page-rank value $\rho_{i}=P_{i}^{\tau }$ (where *τ* is the time when steady state is reached) for further computation.

$$\begin{array}{c} P_{i}^{t+1}-P_{i}^{t}\leq \epsilon \end{array}$$ (2)

The second stage of computation involves distributing this page-rank value among the respective authors of each publication. The page-rank value of the publication could be distributed equally between the authors. However, we find this distribution to be typically flawed because equal weight would be given to the first author and the last author. However the usual practice is to order the authors by the contributions they made to a certain publication. Hence, in order to maintain fairness and objectivity, we distribute the page-rank values proportionately as shown in Eqs (3) and (4), where $W_{d}^{s}$ is the weight (proportion) of the pagerank value assigned to a particular author (scientist) *s* from document *d*, $N_{a}^{d}$ is the number of authors of document *d*, whereas *R*_*s*_ is the ‘position’ of author (scientist) *s* in the list of authors in document *d*. Further, *ρ*_*d*_ is the pagerank value of document (node) *d* *at steady state*, and $\rho_{d}^{s}$ is the pagerank value assigned to author *s* from it. For example, if a document had five authors, the denominator of Eq (3) would be 15, and the first author would receive a portion of 5−1+1/15 = 5/15, and the second author would receive 4/15 and so on, so that the last author would receive 1/15 of the pagerank ‘value’ of document *d*. A similar scheme has been used to ‘weigh’ author contributions in [18]. It is easy to see that lower ranked authors in a long list of authors are heavily penalised by this system, which would ensure that ‘piggy-bagging’ isn’t rewarded.

$$\begin{array}{c} W_{d}^{s}=\frac{N_{a}-R_{s}+1}{0.5N_{a}\left(N_{a}+1\right)} \end{array}$$ (3)

$$\begin{array}{c} \rho_{d}^{s}=W_{d}^{s}\cdot \rho_{d} \end{array}$$ (4)

We believe it is the general practise to order authors by their level of contribution as suggested by [19] and [20] backed up by empirical evidence. Indeed, [20] suggests that more than 80% of papers published today confirm with this practise and the scientific world is converging towards it. However some disciplines indeed still list authors in alphabetical order. If the pagerank-index is used for analysis exclusively in such a discipline, it could be implemented in such a way that the pagerank-values are distributed equally among authors. Similarly, Hu et al. argues that in some fields, the last author, being a senior colleague, often plays a critical role [21]. In such fields, the weighting scheme given by Eq (3) could be appropriately modified to reflect this. Therefore it is important to emphasise that the proposed pagerank-index does not depend on Eq (3) and this equation could be easily modified to suit the needs of the field or database in which the index is applied. In this pilot study, though, we simply use one model given by Eq (3) rather than focussing on diverse models for author contribution weights.

The final stage involves aggregating the page-rank values received by each author from each of their publications respectively, to come up with a single page-rank summation value for each author node in the collaboration network, as shown in Eq (5). For simplicity, henceforth we will call this the ‘pValue’ Ω_*s*_ of each scientist *s*, with the understanding that it is not computed by running pagerank on collaboration networks, but rather by running pagerank on citation networks and computing a weighted sum, as described above, for each author. Like the page-rank value in citation networks, this is also a decimal value. Therefore, for intuitive interpretation, we then calculate a percentile ranking based on the ‘pValue’ Ω of each scientist. It is this percentile score that is named the ‘**p**agerank-**i**ndex’ *π* of a scientist, as shown in Eq (6). Hence the pagerank-index will not only be a measure of a researchers’ scientific output, but it also will tell where exactly a researcher stands among his peers in a particular scholarly database/field.

$$\begin{array}{c} \Omega_{s}=\Sigma_{d}\rho_{d}^{s} \end{array}$$ (5)

$$\begin{array}{c} \pi_{s}=\text{percentile}\left[\Omega_{s}\right] \end{array}$$ (6)

If the citation network we used is for a specific field, then the derived pagerank-index would be valid for that specific field. Therefore a researcher can have multiple *π* values, and some of these can be more ‘generic’ than the others, including a pagerank-index is computed over the whole scientific community (like the h-index is). However, in practise this ‘overall’ pagerank-index would be limited by the outreach of the specific database. Yet, this is also the case for existing measures such as h-index. For instance, a researcher who works in Machine Learning can have a pagerank-index for machine learning, a pagerank-index for artificial intelligence, a pagerank-index for computer science and an ‘overall’ pagerank-index in the scientific community. This multiplicity makes it easier for pagerank-index to be used to compare researchers. For example, it is well known that it is more difficult to be cited in some fields compared to others, and researchers from these fields complain that they as a result get lower h-indices. However, if a field-wise comparison is made using pagerank-index, leading lights in each field would have high pagerank-index, while pagerank-index can also be used generically. Thus pagerank-index gives flexibility in usage and we consider this to be an important advantage of pagerank-index over existing measures.

It should be noted that ideally, pagerank-index of all authors needs to be recalculated every time a paper is added to the database, since unlike h-index, the addition of a paper might affect any or all authors in terms of pagerank-index. Since this may not be practical, a decision should be made about the periodicity with which pagerank-index is re-computed. For example, the underlying citation network could be updated and page-rank algorithm could be rerun every hour, every day, or every week. This would be a parameter of the system, but in a typically large database, the disadvantage an author may suffer due to pagerank not being run immediately would be minimal and temporary. In any case, compared to the time it often takes for a document to be picked up by a database such as Google scholar after it becomes available in the web, the time it may take for it to influence the pagerank-index will be minimal.

## Analysis

### Demonstrating the utility of pagerank-index using synthesized systems of scientists

We have stated that the pagerank-index is ‘fairer’ to the scientists compared to the existing metrics, and that it can help minimise the impact of massaging and potential for questionable practises. In this section we demonstrate this by developing a synthesized system of scientists working in an evolving field, and ‘running experiments’ on this system. This approach is essential since we cannot control, or cast aspersions about, the intentions or behaviour of authors in real world databases. Thus a simulated system is best to test the proposed index. The system simulates the process whereby a group of scientists produce papers in collaboration and this spawns a new field of research. Eventually, this gives rise to a citation network, where papers are nodes and citations are links, as well as a collaboration network where the scientists are the nodes and collaborations (co-authorships) form the links. The former are directed networks, and the latter are undirected. As described below, the evolution process realistically imitates the growth of a research field and corresponding citation and collaboration networks.

We consider three independent ‘case studies’ using this synthesized system. Each case is meant to illustrate a perceived weakness of h-index, and how the pagerank-index is relatively immune to it. In each case or scenario, two ‘groups’ of authors are contrasted based on an inherent publication habit in which they are different. The authors are assumed to be otherwise similar, on average, between the two groups. The intent is to show that while h-index is heavily biased towards one group, the pagerank-index rectifies this imbalance.

In our synthesized system, there are paper objects and author objects. Each of these objects have a number of variables (attributes). These attributes are summarised in S1 Appendix with a short description of each attribute. To minimise technical details, we avoid going into the software design aspects here, except to state that the variable **Member** is a ‘generic’ attribute, and takes on different names and meanings in each simulation scenario described below. It essentially indicates whether an author/paper belongs to a certain group or the ‘other’ group in that scenario.

The system simulates the evolution of a field in the following manner. At the beginning of the simulation, a sequence of papers are ‘spawned’ and a certain number of authors *N*_*a*_ are spawned and assigned to each paper. The number of authors *N*_*a*_ is a random number between 2–5. Each spawned paper is also randomly assigned an impact factor between 0–20. For simplicity, in this initial study we keep the distribution of impact factors linear, though clearly it is biased towards lower values. We also assume that conference papers and similar documents have zero ‘impact factor’, and thus are generally regarded lower than journal papers. When papers after the first paper are spawned, a certain number $N_{a}^{*}\leq N_{a}$ of authors are randomly selected and assigned from the existing author pool, while the rest $N_{a}-N_{a}^{*}$ authors are newly spawned. This represents new authors being introduced to the field as ‘collaborators’ of existing authors, as it typically happens. The probability of new authors being spawned decreases as the simulation progresses, so that authors are increasingly assigned from existing author pool. At steady state, the probability of an author being a new author rather than somebody from the existing author pool was fixed at 4%. Each spawned paper is randomly assigned a number *N*_*r*_ between 10 and 50 to represent the length of its reference list, since 10–50 references are typical for most scientific papers. However, not all of these references are necessarily to existing papers in the simulated system. In real world, a paper which is a pioneer in a new field will by necessity refer to papers ‘outside’ its field, since there are no papers already in the emerging field to cite. Even in a saturated field, a certain proportion of the references will be outside the field anyway. To reflect this, we set proportion *ψ* as the proportion of references which are within the ‘field’, and this proportion begins with zero and increases linearly with each paper until it reaches the steady-state value of 70%. Thus, the number of ‘citations’ by each paper to other existing papers within the field is *ψN*_*r*_. Once the number of internal references is determined, existing paper objects are chosen to be the references in the newly spawned paper. These are chosen by weighted preferential attachment, as described below. Thus, the more a paper is already cited, the higher its chances are to be cited by a newly spawned paper. This is done because in real world also, already highly cited papers are more likely to be noticed and cited by new papers.

The references by newly spawned papers to the pool of existing papers were made by using the well-known ‘preferential attachment’ method. We use a version introduced by Albert and Barabasi [22], though it is suggested by some scholars that the concept of preferential attachment has been introduced much earlier by Herbert Simon [23], and Price [24] has in fact used it in analysing citation patterns as early as 1976. A weighted preferential attachment scheme was used, so that existing papers have a higher chance of being cited by newly spawned papers if they had already a high number of citations or published in a journal of high impact factor. It was ensured that papers which were never cited or published in an outlet of zero impact factor could still be cited, albeit with a very small probability. Eventually, a directed citation network and an undirected collaboration network begin to evolve. We continue this process for a fixed number of ‘timesteps’. Timesteps have no meaning in terms of physical time but simply denote an instance where a new paper is spawned. In this paper, typically *T* = 25000 was used. In our experiments, we maintained the number of papers: number of authors ratio at roughly 10:1.

After each timestep, the pagerank algorithm was run on the citation network *until steady state*, and the pagerank score, and by extension the pagerank-index, of authors were updated. The h-index of authors was also updated, using the available local information for each author (list of papers and citations for each paper). As such, we were able to compare the evolutionary trends of h-index and pagerank-index for each author. Our results are presented below.

#### Measures and plots

The following plots and metrics are used to present the results of the simulation experiments.

- Plots of the distribution of the h-index and pagerank-index: These plots are used to visualise the disparity between the two indices for each author at a given point in time, typically at the end of simulation. The h-index and pagerank-index are plotted against author ID.
- Plots of the time variation of average h-index and pagerank-index: Average values of the h-index and pagerank-index for a particular ‘group’ of authors are plotted against time.
- Plots of the time variation of Maximum h-index and pagerank-index: Highest (best) values of the h-index and pagerank-index among a particular ‘group’ of authors are plotted against time.

In the next subsections, we describe the three simulation scenarios we have undertaken. Each scenario aims to highlight a suspected or known weakness of h-index, and how pagerank-index could address this. To do so, we typically spawn two groups of authors, whose behaviour patterns are different in a given crucial aspect, which may give one group an advantage over other in terms of h-index, and we consider how this advantage is negated when pagerank-index is used.

#### Scenario 1: The impact of self-citing low impact publications

The first simulation scenario demonstrates a known weakness in h-index where certain authors can potentially publish low impact papers in order to cite their previous work and thus ‘massage’ their h-index. We intend to demonstrate how the pagerank-index copes against such a scenario. Therefore, in our simulation system we spawn two groups of authors, with one group having an inherent ‘tendency’ to massage their h-index this way, while the other group does not. We will call these groups ‘manipulative authors’ and ‘non-manipulative authors’ for ease of reference henceforth, with the understanding that only some papers spawned by ‘manipulative authors’ are written to massage citations, while others are genuine papers. We assume that all papers spawned with the intention of massaging citations are low-impact documents, since the authors have no incentive to bother with the quality of the output in such scenarios. We will refer to those documents which are created purely to massage citations as ‘manipulative documents’. A manipulative document, once spawned, will be assigned authors only from the ‘manipulative authors’ pool. A paper is randomly determined to be a manipulative document with a probability of 0.1 at the time of creation. The generic variable name Member is modified such that the Member parameter for paper nodes will be named as **isManipulativePaper** while the Member parameter for authors will be **isManipulativeAuthor** in this scenario. The probability of being a manipulative author is set to 0.2 and each new author, when created by the simulation system, will be randomly assigned as either manipulative or non-manipulative. Both non-manipulative and manipulative authors can be assigned as authors of non-manipulative papers. The sole purpose of a manipulative document is to cite the authors’ previous work thereby massaging their h-index. While this is an extreme scenario which rarely happens in real world, it is suitable to demonstrate the effect of such documents in the h-index and pagerank-index easily.

We ran simulation experiments up to *T* = 25000 (i.e created a citation network of 25,000 documents). Even though we ran multiple simulations to verify our results, ‘averaging’ does not make much sense in understanding the citation count of author communities, and therefore we describe a single simulation and the results of it here. This simulation had 2155 authors at end of simulation (*T* = 25000) among which 252 authors were designated manipulative authors and 2500 documents were designated manipulative documents. A snapshot of the resultant collaboration network is presented in S1 Appendix (Some topological properties of the citation and collaboration networks from this simulation run are shown in tables and figures in S1 Supplementary Material. Interestingly, the citation network shows power-law characteristics, whereas the collaboration network does not show any such characteristic).

**Fig 3:**
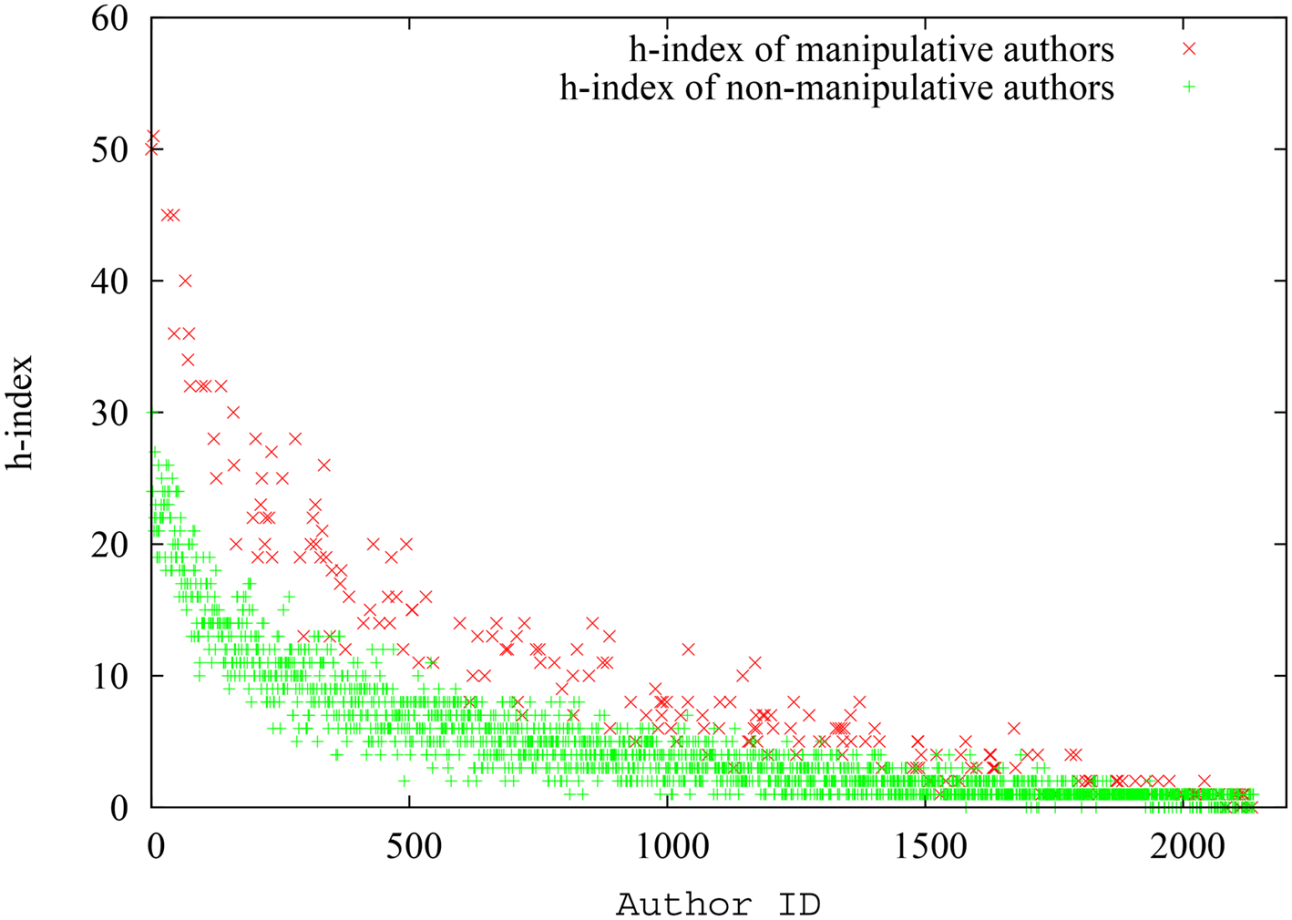
Spread of the h-index for each manipulative and non-manipulative author (as absolute values) in the first simulation scenario. For authors of similar seniority, the ‘manipulative’ author group has a clear advantage.

**Fig 4:**
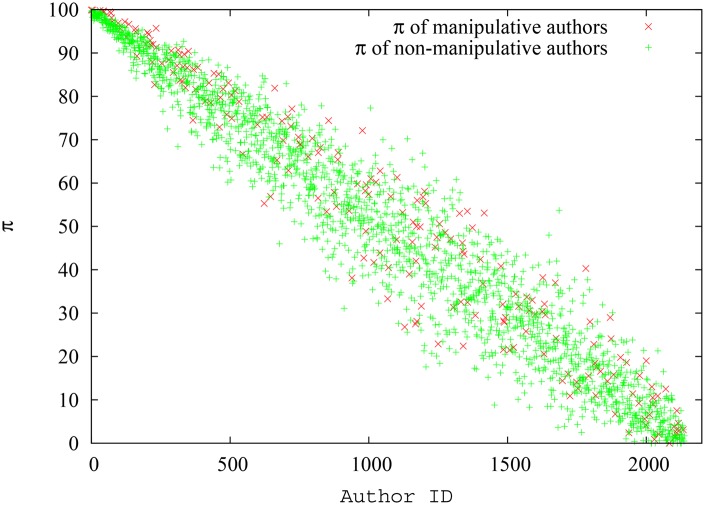
Spread of the pagerank-index for each manipulative and non-manipulative author (as absolute values) in the first simulation scenario. Neither group of authors have a clear advantage here.

The manipulative authors had 70.6 papers per author on average and 256.2 citations per author on average, while the corresponding numbers for non-manipulative authors were 35.4 and 130.2. Therefore, it is clear that the manipulative authors had a clear advantage in terms of paper and citation counts. Indeed, as portrayed in Fig 3, the spread of the h-index for non-manipulative authors and manipulative authors provide evidence to the fact that manipulative authors can indeed massage their h-index by publishing low impact papers with the sole purpose of referencing their previous work. Though the later-spawned authors (authors with higher IDs) naturally have lower h-index, the h-index of manipulative authors is consistently higher than that of non-manipulative authors across the x-axis. On the contrary, we can detect a mixed distribution for the pagerank-index of manipulative and non-manipulative authors as seen in Fig 4. Though again the pagerank-index increases with seniority in general, there is no demonstrable separation between manipulative authors and non-manipulative authors, despite the fact that the pool of manipulative authors, in this experiment, have an extra set of papers that are used exclusively to cite themselves! This is quite remarkable.

**Fig 5:**
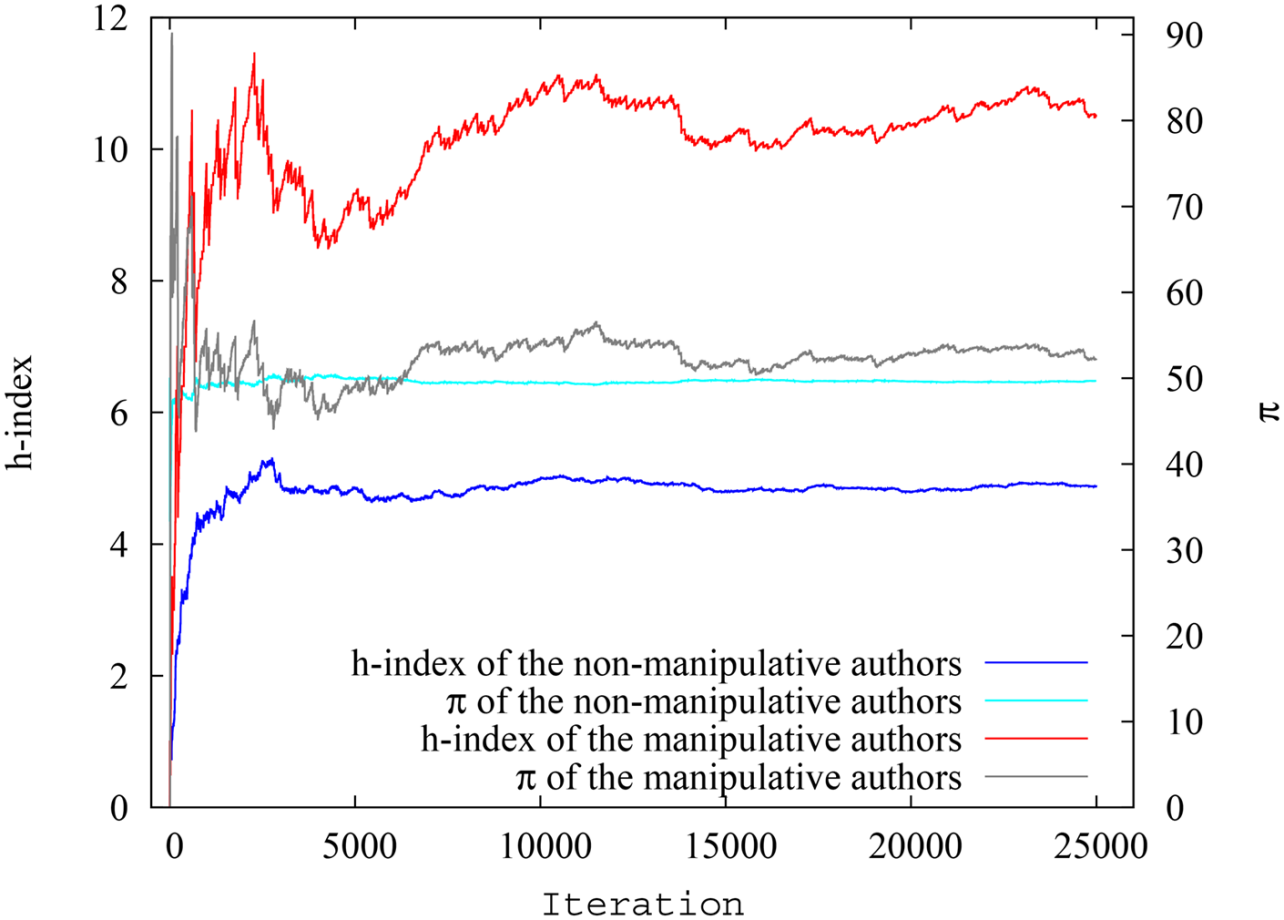
Variation of average h-index and pagerank-index for non-manipulative and manipulative authors at each timestep for simulation scenario 1. The difference is much smaller between the two groups when pagerank-index is considered.

Variation of the average h-index and pagerank-index for non-manipulative and manipulative authors against time is shown in Fig 5. It can be inferred from Fig 5 that pagerank-index is a fairer metric to rank scientists, since the difference between manipulative and non-manipulative authors is much smaller when pagerank-index is used. Quantitatively, at the end of simulation, the difference of h-index is 108.64%(That is, compared to the average h-index of non-manipulative authors, the manipulative authors have a 108.64% gain. The gain is similarly expressed in percentages throughout the paper, even when percentiles are used.) whereas the difference of pagerank-index is only 9.92% and is ten times smaller. The implication is that using h-index, a non-manipulative author will be penalized in average about ten times more than using pagerank-index (Since pagerank-index is a percentile, we also tested whether the percentile ranking of pagerank-index makes the difference smaller (not shown). Thus, we converted the h-index of each group of authors into percentiles, and verified that even here, pagerank-index still emerges as the fairer index but the advantage of using pagerank-index diminishes slightly when a percentile h-index is used. At the end of this particular simulation, the difference of h-index percentile is 52.77% while the difference of pagerank-index is 10.22%). We also can see that the difference between pagerank-index and h-index is relatively stable against time after the initial stages of community growth. Therefore, as Fig 5 shows, the pagerank-index is indeed a fairer measure in comparison to h-index in reducing potential manipulation by authors who may self-cite. Even though we have chosen extreme self-citation where some documents are created exclusively for self-citation, the decisive results indicate that they would be true in milder cases of self-citation or ‘group citation’ (where a particular group of authors cite each other in a circular fashion) as well. The very nature of pagerank-index calculation means that creating documents which do not have ‘wide and deep’ impact is not going to help the authors.

**Fig 6:**
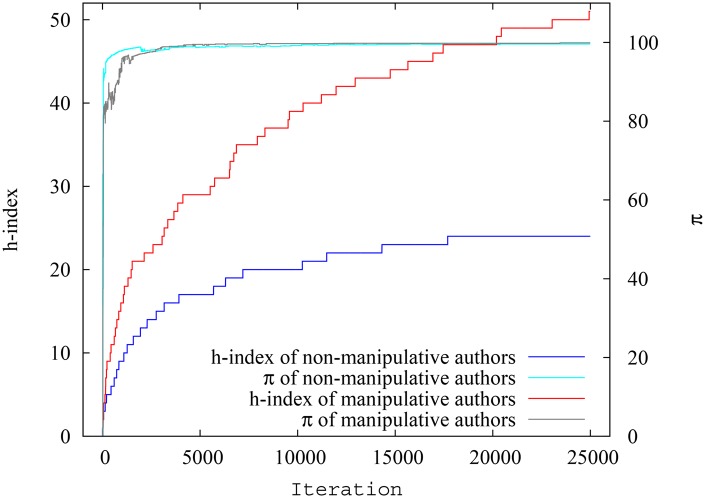
Variation of h-index and pagerank-index for highest ranking non-manipulative and manipulative authors at each timestep for simulation scenario 1.

Fig 6 shows the h-index and pagerank-index respectively for the *highest performing authors* belonging to each group in the system, at each particular timestep. Here the difference between the indices is ever starker. The pagerank-index difference between non-manipulative and manipulative authors is insignificant compared to the h-index difference: 0.54% and 126.2% respectively, at the end of the simulation.

#### Scenario 2: The impact on ‘singleton’ authors

In the second simulation scenario, the intention is to demonstrate that pagerank-index is fairer to authors who are less inclined to collaborate. It is intuitively understood that the more a scientist collaborates, the higher the number of papers he can get, and the higher the number of citations that will follow. For example, let us say hypothetically that a scientist spending two hundred hours can write a full paper (including design, experiments, and writing), and that a scientist has six hundred hours per year to do research, and a paper gets fifty citations per year in her field. A scientist working on her own should be able to produce three papers, which should fetch her 150 citations after one year. A scientist collaborating with another one, where they share the workload equally in each paper, however would be a co-author in six papers and thus get 300 citations after one year. Their h-index values would be affected accordingly. While this example is too simplistic, it is obvious that the h-index of a more collaborative scientist would typically be higher, even if the level of expertise, amount of work done, and originally etc are equal among two scientists. We now analyse up to what extent the pagerank-index can correct this imbalance.

Therefore, in the simulated system we divide the authors into two groups again: authors who have an ‘inherent’ tendency to collaborate, and authors who do not have this tendency. The ‘collaborative’ authors would typically publish documents with 1-9 authors per each paper. The second group of authors is relatively not interested in collaboration and would only publish papers that have three authors at most. The system would spawn two types of papers accordingly: collaborative papers and non-collaborative papers, which would be assigned authors only from the respective relevant pool of authors. The generic Member parameter here is named *isCollaborativeAuthor* and *isCollaborativePaper* respectively for author and paper nodes(Please note, however, that being ‘collaborative’ is not an inherent tendency of the paper: this property is given to a paper node just so that it could be assigned to the relevant author pool). Collaborative authors in this scenario consist of 90% of the author pool while 10% of authors are non-collaborative authors. The publications are assigned so that 80% are collaborative publications and 20% are non-collaborative publications.

Again, even though we simulated the scenario several times, we present the results of one run since averaging is meaningless, though we have verified that the results presented below are typical. In this run, the system was simulated until 25000 papers were spawned consisting of 5012 non-collaborative papers. The author pool at the end of simulation included 2215 authors with 201 non-collaborative authors in total. The collaborative authors had 104.8 papers per author on average and 82.4 citations per author on average, while the non-collaborative authors had 13.2 papers per author on average and 11.8 citations per author on average. Note that the collaborative authors, by virtue of extensive collaboration, have a massive advantage in terms of average paper and citation counts(The topological characteristics of the evolved citation network and the collaboration network at the end of simulation are included in S1 Supplementary Material. We may again note that the citation network displays scale-free characteristics, while the collaboration network does not).

**Fig 7:**
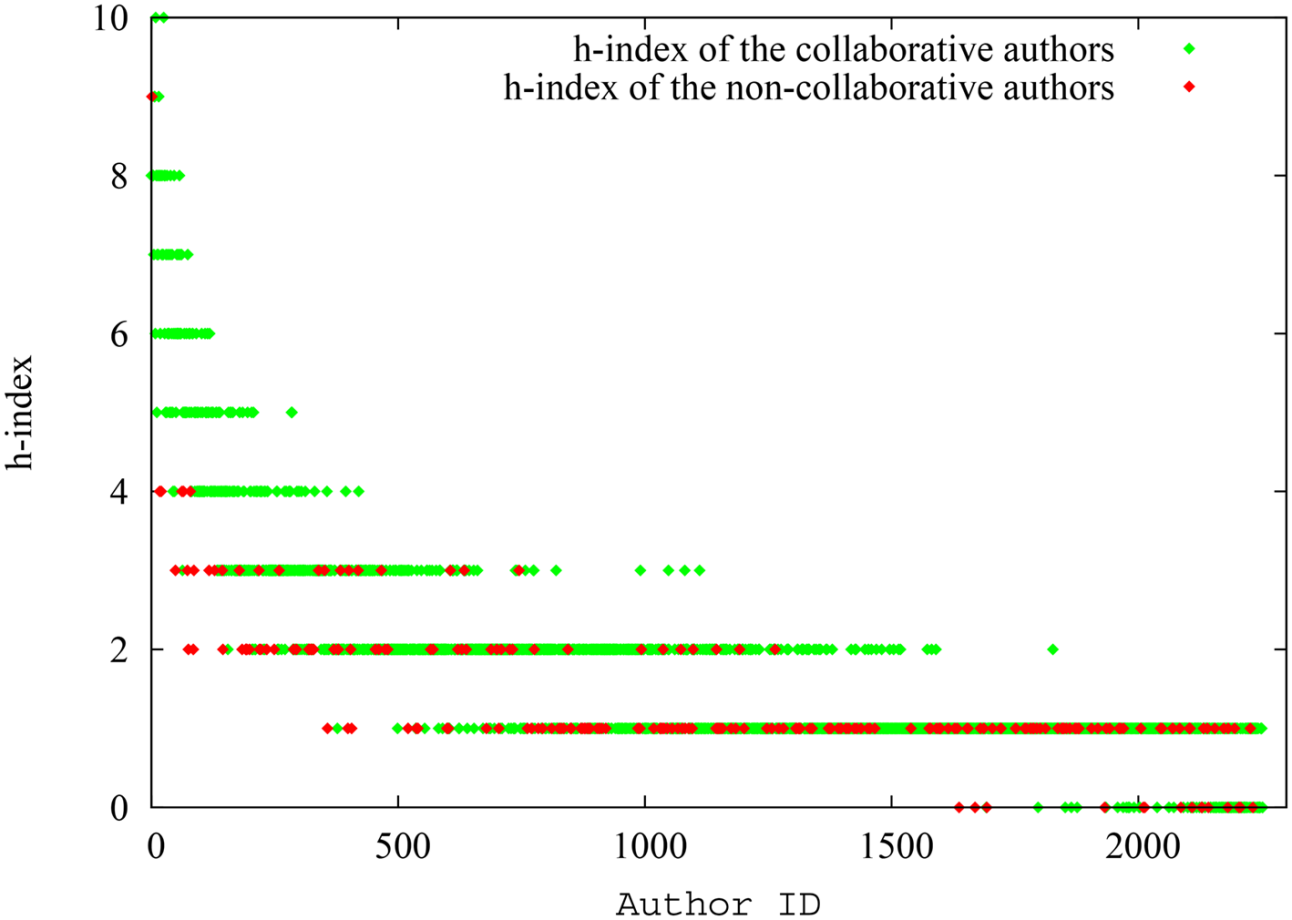
The spread of h-index for collaborative and non-collaborative authors (as absolute values) in scenario 2. For authors of a similar level of seniority (as indicated by their IDs), the ‘collaborative’ authors have a clear advantage.

**Fig 8:**
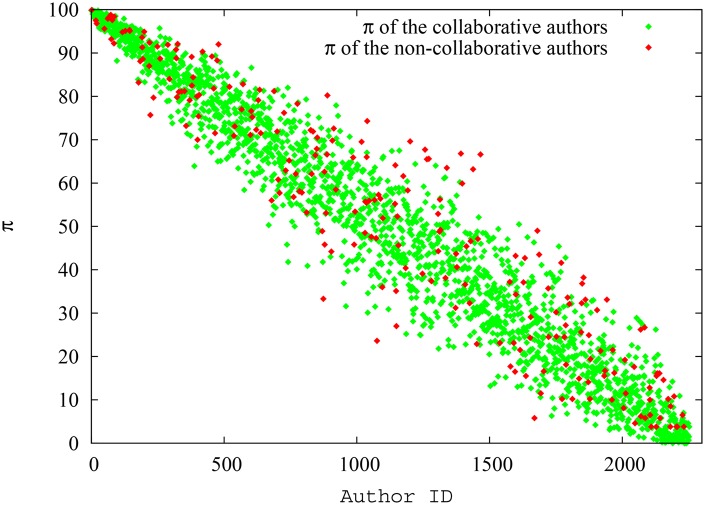
The spread of pagerank-index for collaborative and non-collaborative authors (as absolute values) in scenario 2. No group of authors have a clear advantage over the other group.

**Fig 9:**
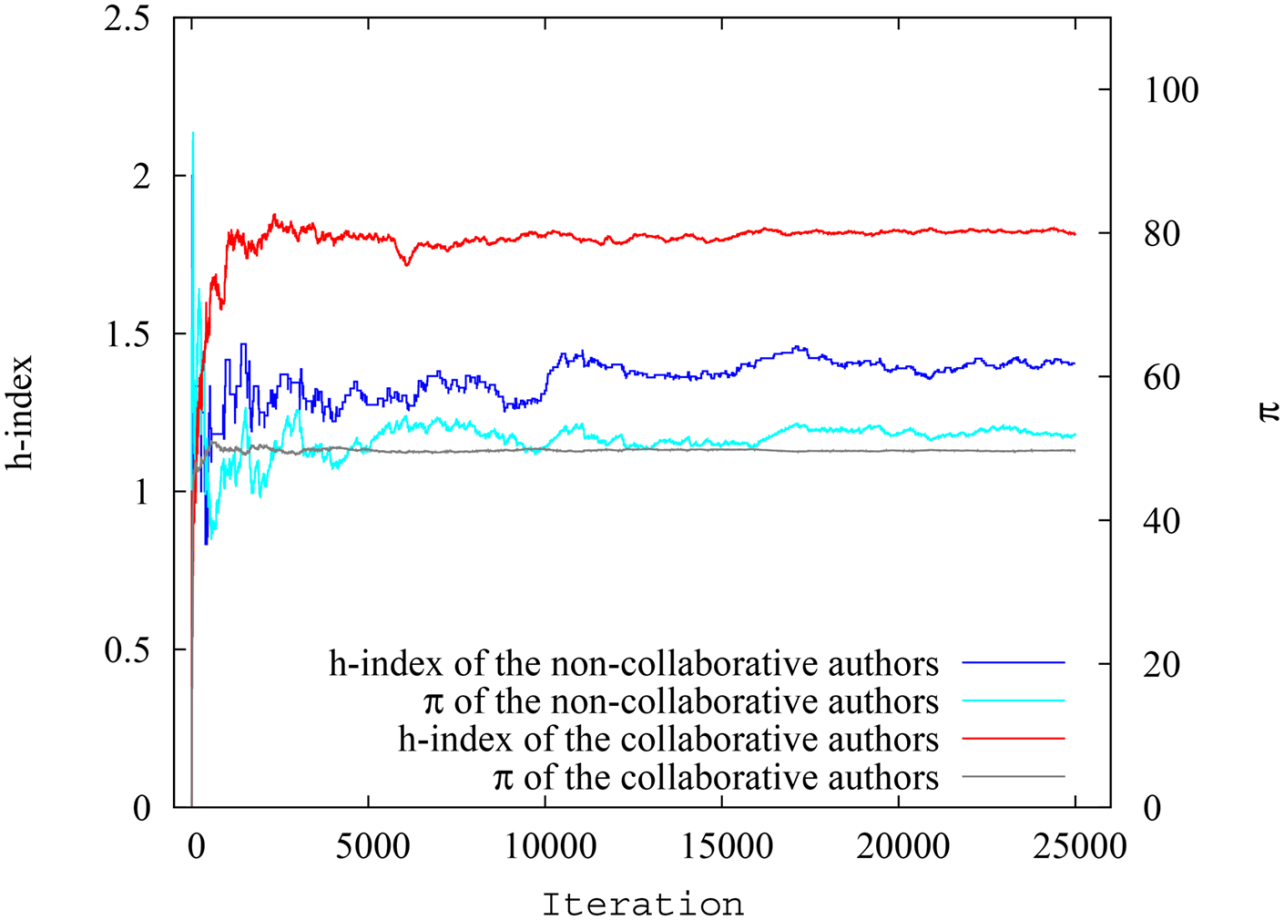
Variation of average h-index and pagerank-index for collaborative and non-collaborative authors at each timestep in simulation scenario 2. The difference between groups is much smaller when pagerank-index is used.

**Fig 10:**
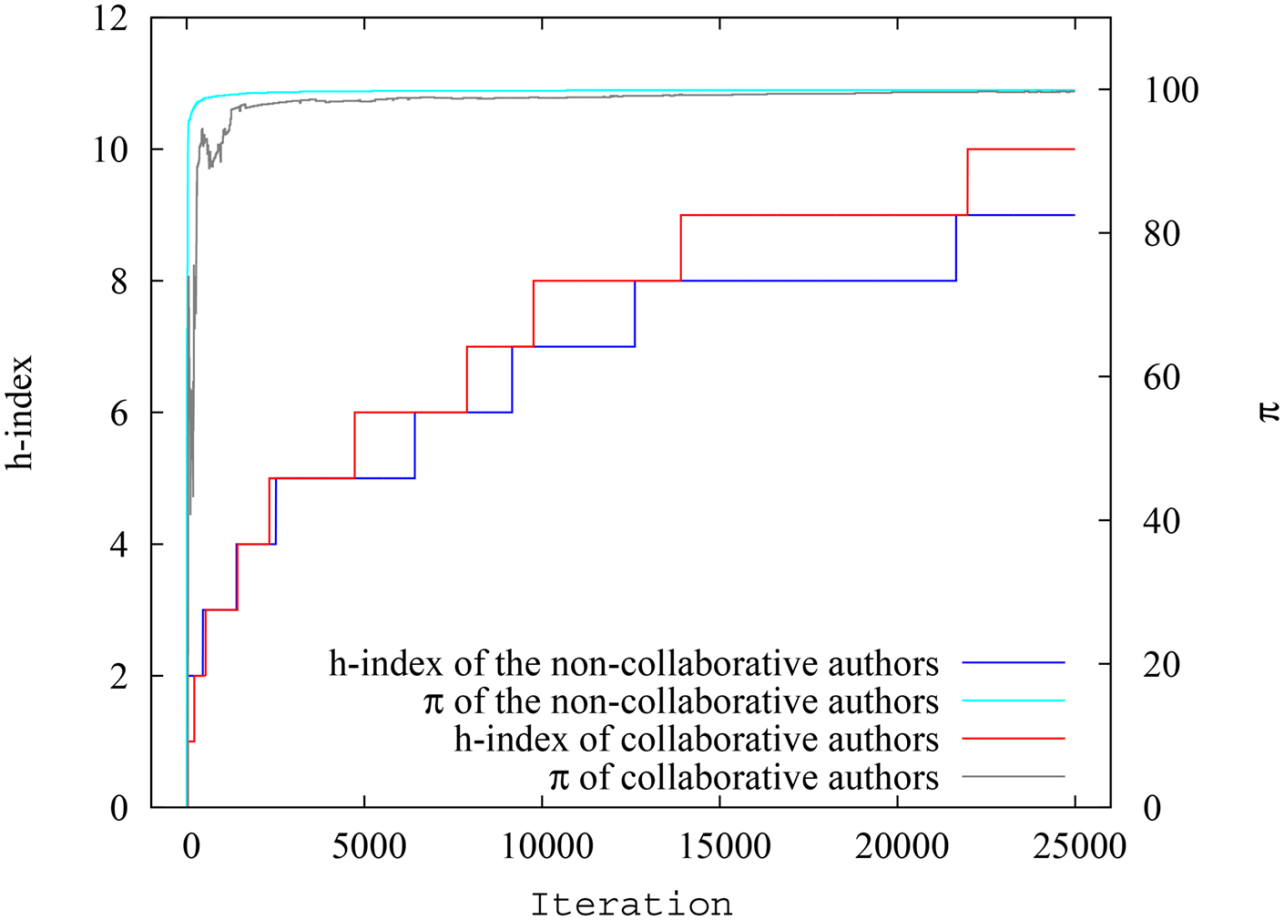
Variation of h-index and pagerank-index for highest ranking collaborative and non-collaborative authors at each timestep in simulation scenario 2. The difference between groups is much smaller when pagerank-index is used.

We are interested in the h-index and pagerank-index spread for collaborative and non-collaborative authors. The Fig 7 shows the spread of h-index for collaborative and non-collaborative authors, while Fig 8 shows the spread of pagerank-index for collaborative and non-collaborative authors, against author ID. As seen in the previous scenario, the seniority-related effects not withstanding, we may see that most non-collaborative authors have relatively low h-indices, whereas in terms of p-indices the distribution is more evenly spread. Analyzing the average fluctuation of h-index and pagerank-index for collaborative and non-collaborative authors reveal interesting characteristics. As shown in Fig 9 which plots the average h-index and average pagerank-index against time for each group of authors, average h-index of the collaborative authors is 32.4% higher compared to non-collaborative authors, while average pagerank-index difference is only 1.52%. From Figs 7, 8 and 9 we can infer that pagerank-index is much more robust to individual tendencies to collaborate extensively. To further validate this, the h-index and pagerank-index of the highest ranking authors from each group at a given time are plotted in Fig 10. This plot re-emphasizes that neither class of authors would gain a quantifiable benefit by using pagerank-index whereas it is entirely possible to gain such a benefit by using h-index. Again, the collaborative tendencies we have considered are ‘extreme’, however this experiment highlights how pagerank-index would negate the unfair advantage some authors can gain by using extensive collaboration. When pagerank-index is used, it matters less how one’s time is divided: whether one focuses all one’s time in one paper or splits it into three, one is nearly equally rewarded as long as the quality of the work remains the same. That is how it should be.

#### Scenario 3: Handling the balance between quality and quantity of publications

The third simulation scenario deals with a typical problem scientists face when being ranked by traditional indices such as the h-index. Intuitively, if a scientist is more concerned about the quality of his publications rather than quantity, we understand that his h-index might suffer as a result, and this has been observed in real world by studies such as by Van Raan [9]. Even though it could be argued that high-quality publications are likely to result in higher number of citations, ultimately the h-index is also bound by the number of papers a scientist has produced. For example, a scientist who has written only ten papers in his life time cannot have an h-index greater than ten, even if each of these papers have been cited a thousand times. This typically affects scientists of a bygone era, where number of papers each scientist produced may have been less. However, even in the present era, it could be argued that a scientist maintaining an ‘optimal balance’ between quality and quantity is most likely to be favoured by h-index, though maintaining such a balance does not necessarily make him a better scientist. This issue is not the same as the one described in scenario 2, because even a singleton author may choose to either focus on quality or focus on quantity, though in general there is correlation between the extent of collaboration and the number of papers produced. This scenario, however, considers an author’s tendency to focus on quality as opposed to quantity, regardless of whether that is done individually or in a collaborative setting.

The simulation scenario, as before, involved two types of authors; those who are ‘quality oriented’ and those who are ‘quantity oriented’, these terms being relative. In order to quantitatively justify the impact, the simulations were set up such that the quantity oriented authors publish papers with an impact factor of 0-2 while quality oriented authors publish papers with an impact factor of 0-20. While such ranges may not be realistic, they serve to highlight the difference well. The system was set up to generate roughly 10% of quantity oriented authors with 10% of ‘quantity oriented papers’, which are basically papers assigned exclusively to this author pool. Again, as before, we note that being ‘quality oriented/quantity oriented’ is not and cannot be an inherent property of the paper, and this is a ‘marker’ simply denoting that these papers when spawned will be ‘assigned’ to authors within a particular pool. Again, even though we ran several simulation runs, we present the results of a typical run below since it is unrepresentative to ‘average’ the results.

**Fig 11:**
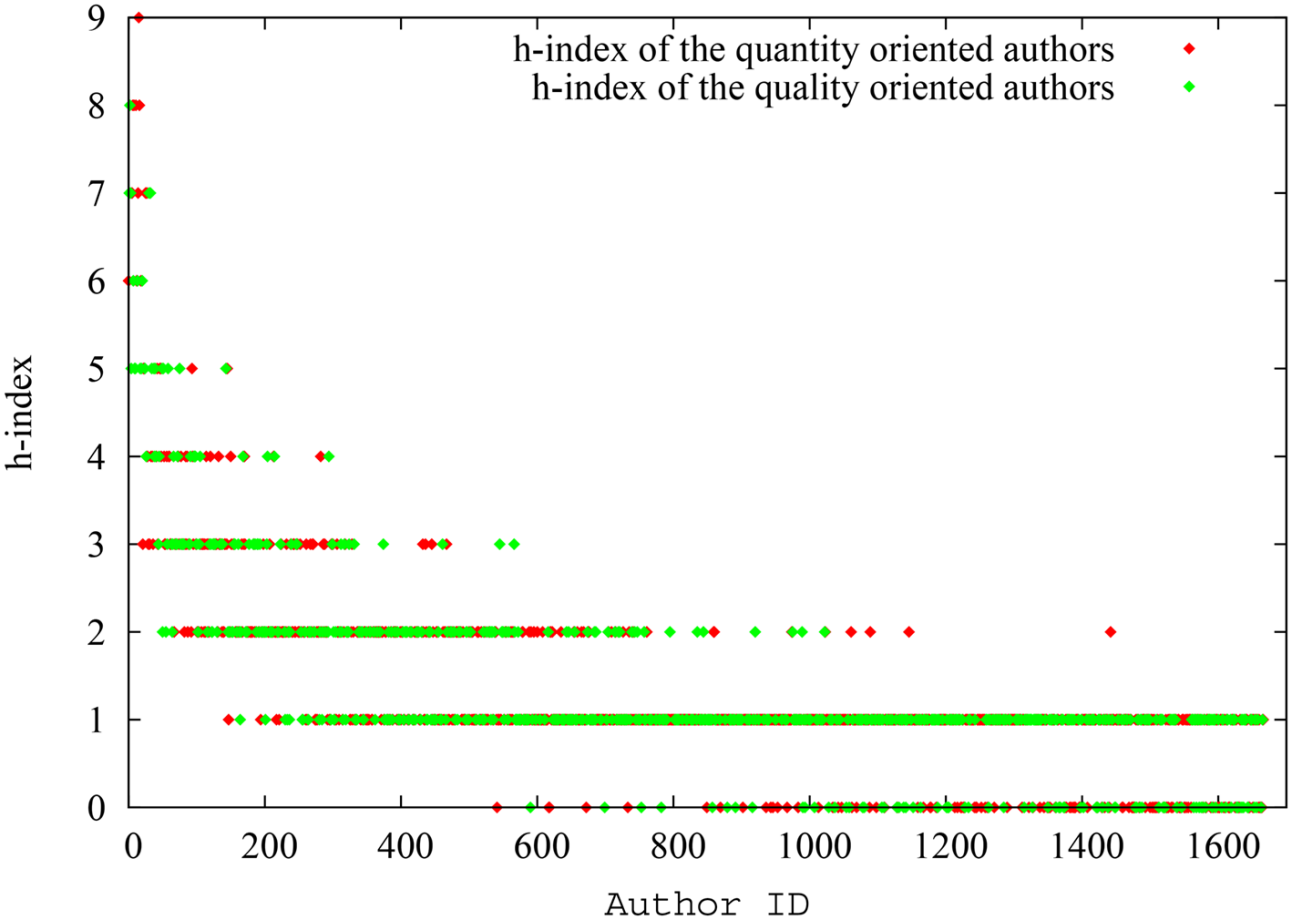
The spread of h-index for quantity oriented authors and quality oriented authors (as absolute values) in scenario 3. Considering authors with the same level of seniority (as indicated by the IDs), the ‘quantity-oriented’ authors have a clear advantage over ‘quality-oriented’ authors.

**Fig 12:**
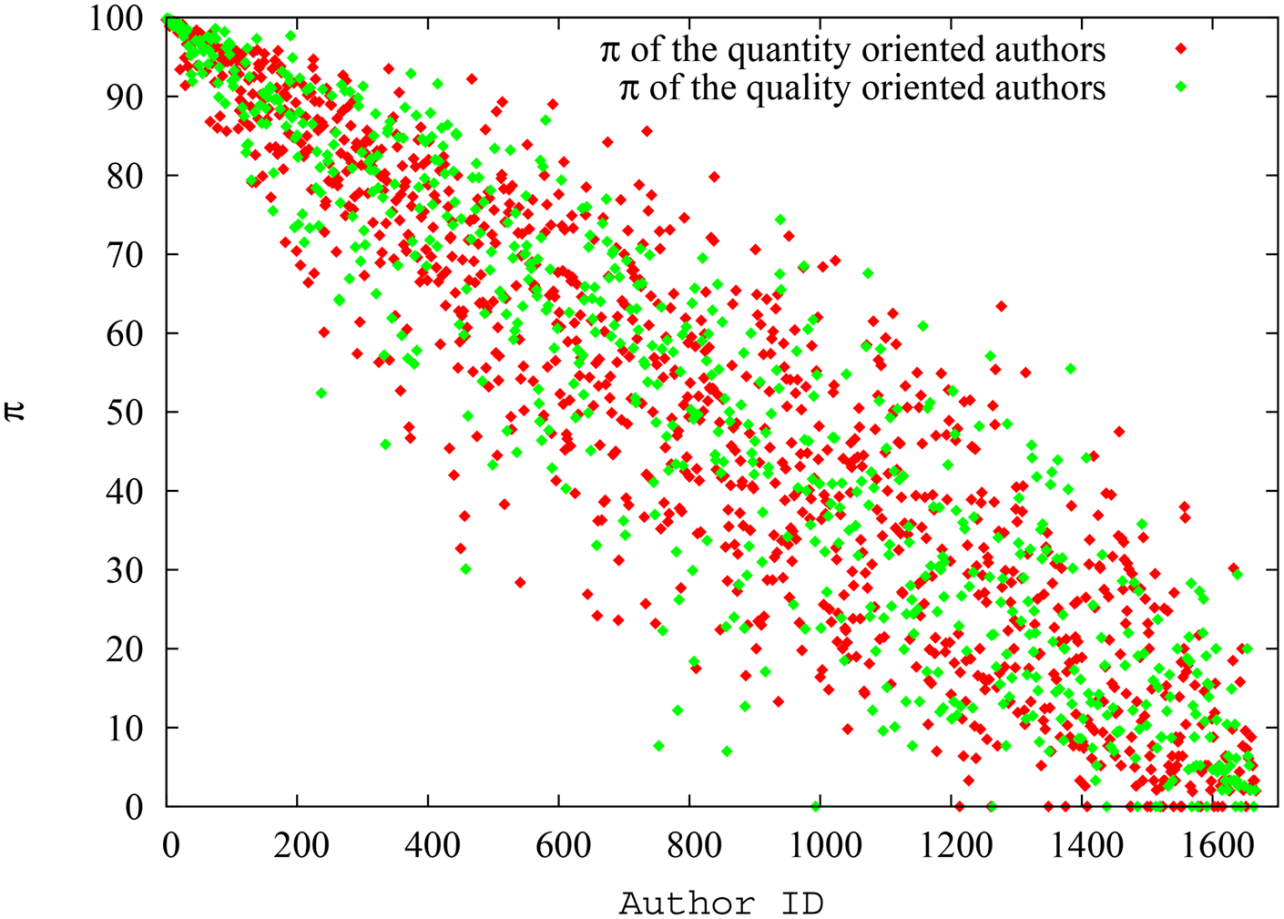
The spread of h-index for quantity oriented authors and quality oriented authors (as absolute values) in scenario 3. No group of authors have a clear advantage over the other group.

**Fig 13:**
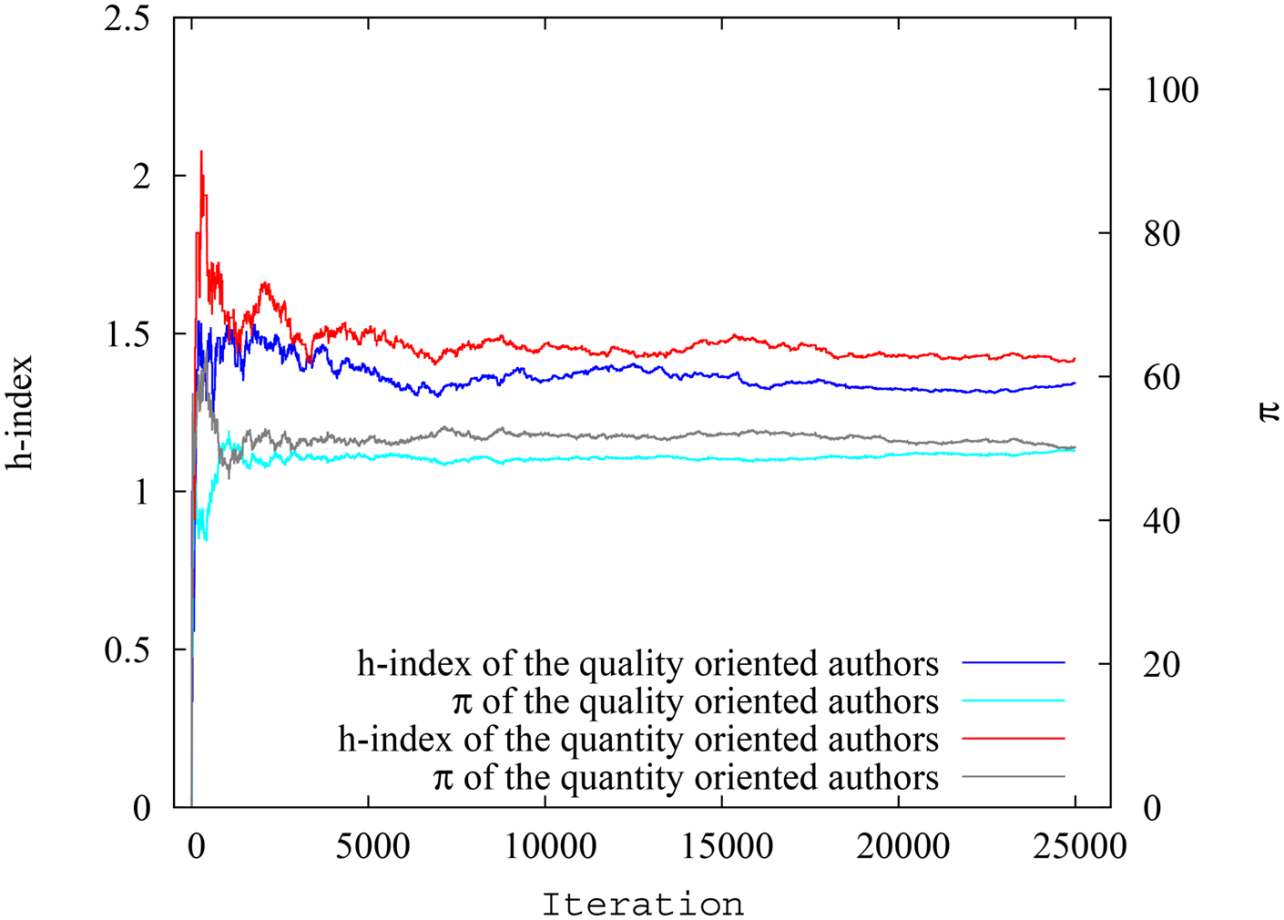
Variation of average h-index and pagerank-index for quantity oriented authors and quality oriented authors at each timestep in scenario 3. The difference between groups is much smaller when pagerank-index is used.

**Fig 14:**
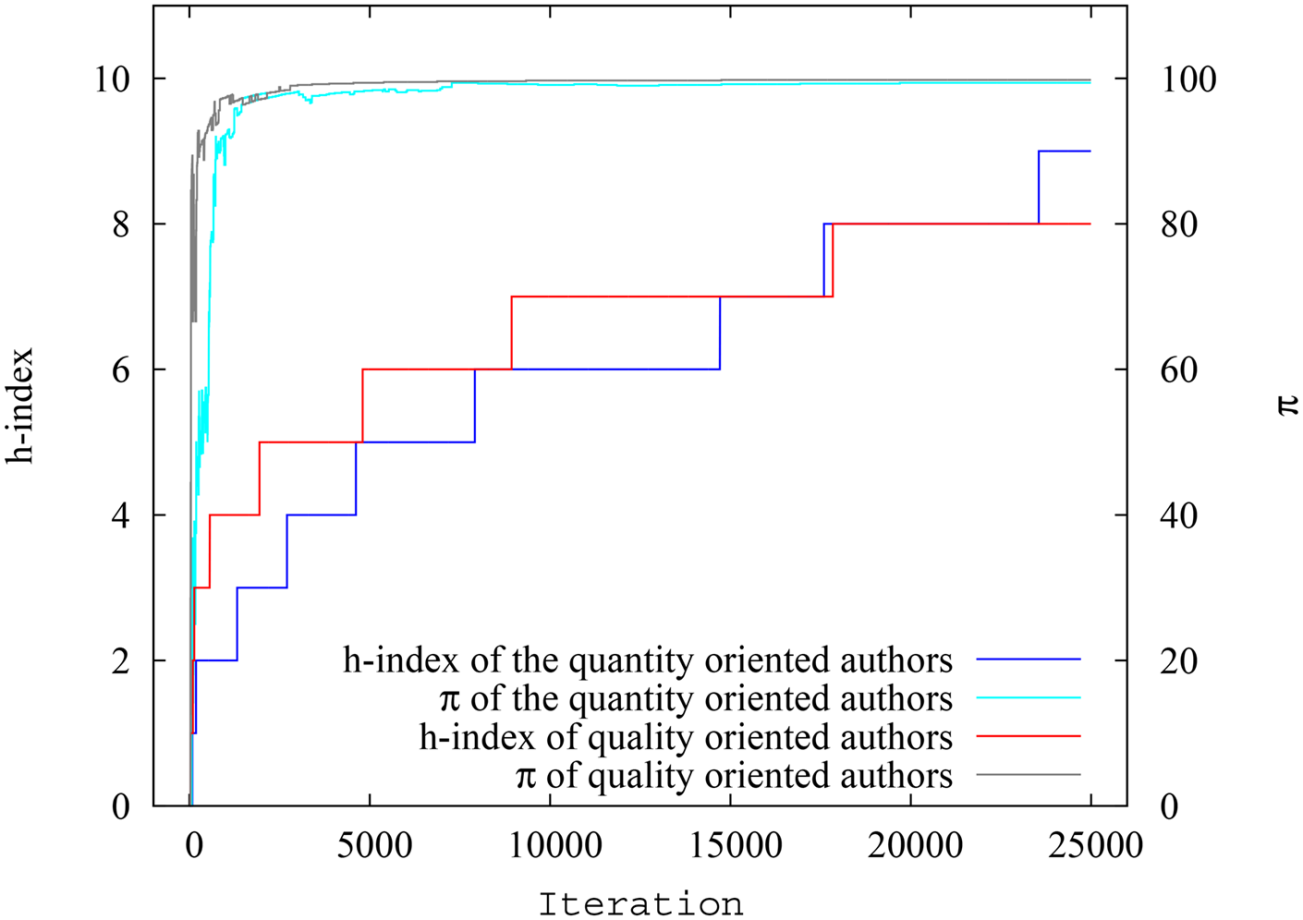
Variation of h-index and pagerank-index for highest ranking quality oriented authors and quantity oriented authors at each timestep in scenario 3. The difference between the highest ranking authors is much smaller when pagerank-index is used.

In this particular run, the system had 25000 papers in total at the end of simulation of which 2524 were ‘quantity oriented’ papers, and had an author pool of 1670 authors with 175 quantity oriented authors (in S1 Supplementary Material, we present the degree distributions for the citation network and collaboration network as well as related properties of these networks. Again, the scale-free nature of the citation network and non-scale-free nature of the collaboration network is obvious). The ‘quantity oriented’ papers, when spawned, where assigned authors only from the ‘quantity oriented’ author pool. However, the ‘quality oriented papers’, when spawned, where assigned authors from either pool, reflecting the fact that even authors who mostly focus on quantity also sometimes could manage to publish high quality papers. In any case, as mentioned before, there is overlap in the impact factor range between both pools of papers. On average, the quantity oriented authors had 39.3 papers and 450.6 citations per author, while the quality oriented authors had 31.3 papers and 126.8 citations per author, so the first group had a clear advantage. In terms of the impact of indices, though, we find similar results as in the previous scenario, showing that the ‘difference’ between the two groups of authors is minimized when pagerank-index is used for comparison. Fig 11 shows the spread of h-index for quality oriented and quantity oriented authors, while Fig 12 shows the spread of pagerank-index for quality oriented and quantity-oriented authors. Again it can be seen that, despite seniority-related variation, the h-index distribution is substantially higher for quantity oriented authors, while the two classes nearly overlap in pagerank-index distribution. Fig 13 shows the variation of the average h-index and average pagerank-index over time of for both groups of authors. From this figure, it can be inferred that average h-index of quantity oriented authors is significantly higher than that for quantity-oriented authors (over 10.4%), whereas average pagerank-index is less affected(about 6.72% higher for quantity oriented authors). Similarly, Fig 14, shows the h-index and pagerank-index values of the highest ranked quality oriented author and quantity oriented author at each time step. Here we can see that the percentage difference of h-index between the ‘best’ scientists in each group is as much as 13.6% while the difference of pagerank-index is as low as 2%. Even though the contrast is less sharp in this third scenario, all of these results indicate that the pagerank-index can reduce the perceived disadvantage ‘quality oriented’ authors may face when their performance is measured by the h-index.

#### Summary of the simulation experiments

The simulation experiments were set up to demonstrate the utility of pagerank-index, and in particular, its supposed advantages over h-index in well known scenarios. The simulation system was set up to reflect the realistic process of evolution of a field where new authors and papers are continually spawned, however extreme scenarios were sometimes used to demonstrate our points clearly, without loss of generality. In each scenario, we studied two groups of authors, who are different by a particular inherent characteristic, with one group supposedly gaining an unfair advantage due to this characteristic. In particular, we considered (i) heavily self-citing authors vs their counterparts (ii) heavily collaborating authors vs their counterparts (iii) heavily publishing authors vs their counterparts. In all three scenarios, we found that a particular group is heavily favoured by h-index, even though it is demonstrably evident that this group is neither more talented nor different in any other way to the authors in the other group, except by the particular inherent tendency we focus on. We also found that by using pagerank-index, we could very significantly reduce this unfair advantage gained by one group, though in most cases the pagerank-index slightly favours the same group heavily favoured by h-index. Yet, the difference is often ten times or more, therefore we can reasonably argue that the pagerank-index is a much fairer measure.

We note here, however that we do not necessarily claim one group of authors are somehow ‘unethical’ in all these scenarios. An author is within her rights to self-cite, and authors who collaborate more or publish a higher number of papers certainly need not feel unprofessional about it. We just observe that the existing measures award unfair advantages to certain inherent publishing habits, which the pagerank-index to a large extent neutralises, and provides a level playing field for all authors regardless of their publication habits. Moreover, the names we gave to various groups of authors, such as ‘collaborative authors’, are only applicable within the context of our own simulations, and to hypothetical authors who have been designed to show extreme behaviour for the purpose of simulation experiments, and not to any groups of authors in real world.

### The impact of pagerank-index in quantifying the performance of scientists in the field of quantum game theory

In the previous section, we demonstrated the utility of pagerank-index within a simulated system of authors and papers. It would be interesting to see what the new index can highlight in a ‘real world’ community of authors. The pagerank-index is meant to be implemented in a large citation database, such as Scopus, ISI Web of Science, or Google Scholar, so that the entire body of scientists listed in that database can be ranked. The h-index, for example, is calculated by many of these databases (such as Google Scholar), even though the h-index displayed in Google Scholar for a scientist does not have to be the ‘real’ h-index of a scientist, and the h-index calculated for the same scientist using different databases could actually be different. It has become the norm to accept the ‘Google Scholar h-index’ of a scientist as the more or less genuine h-index of that scientist. However, to demonstrate the utility of the new index, we need to be able to ‘define’ a particular author community in some way, since the index is not yet implemented in commercial databases. It would be ideal if we can trace the ‘history’ of this field, from its inception. However, this is often not possible. Several journals or groups of journals, such as journals published by APS (American Physical Society) make their citation network database publicly available; while these are large databases, it is cumbersome or impossible to trace the field they represent back to its first paper.

Luckily, some emerging fields of science have dedicated Google Scholar profiles, where authors can voluntarily ‘attach’ their papers. Therefore, it is possible to choose one of these profiles as a ‘field’ for the anaylsis, though such profiles typically do not contain more than a thousand papers and many papers belonging to the relevant field may be missing from these profiles. Such analysis can however be complemented by the analysis of a large dataset publicly available, though it may not have a complete history from the field’s beginning. With these considerations in mind, we choose two ‘real world’ datasets for our analysis: (i) The field of ‘Quantum Game Theory’ as defined by the dedicated Google scholar profile [25] (ii) The arXiv data set of citations representing the field of high energy physics theory [17].

Let us focus on the Quantum Game Theory field in this section. This field (as defined by the Google scholar profile) has achieved sufficient growth to facilitate a meaningful analysis (at time of access, it contained 685 papers, 785 authors, and 3776 citations), at the same time it is sufficiently young such that the growth of the field could be traced back to the ‘first’ papers. Of course, it is conceivable that some papers belonging to this field have not been added to the said profile by the authors. Despite this, the field of quantum game theory as defined by this profile is a suitable dataset to test the utility of the pagerank-index, particularly since pagerank-index of all participating authors could be computed ‘historically’ from the moment the first paper was published.

Therefore we first generated citation networks from this dataset, considering each instance after a new paper has been added as a ‘timestep’. We do not have to consider the time between the addition of two papers, since we only consider ‘internal’ citations, citations from papers in this field to papers in this field. Any citations to or from external papers are ignored. Thus, the addition of a new paper corresponds to a ‘timestep’, and this sense of causality is sufficient for our purposes—we need not worry about the actual dates of publication. To generate citation networks, we assigned IDs to papers then manually prepared a list of references consisting of these IDs for each paper in the field, since reference lists follow various conventions and sometimes do not even include the paper title, and it is difficult to automate this process. Once this manual curing has been done, we generated the citation networks corresponding to each ‘timestep’ using a computer programme, where paper IDs and corresponding reference lists (also in IDs) were inputs (The evolved citation network at four timesteps (*t* = 100,*t* = 200,*t* = 400 and *t* = 685) are shown in the S1 Appendix). The IDs were in the order of publication, therefore to generate a citation network at timestep *t* = *t*_0_ we simply considered all IDs less than *t*_0_. We ran pagerank on these citation networks until steady state to compute the pagerank-index for all authors at each timestep, and we also computed the h-index by considering the total number of citations each author had at a given timestep. Below we discuss the results obtained.

First, let us make some qualitative observations which illustrate the utility of pagerank-index and how it can help rectify biased perceptions of the scientific contributions of scientists in a field. In doing so, let us emphasize that it is not our intention to judge any scientist or their contributions to science: nor are we claiming that the pagerank-index is the ultimate tool for measuring scientific brilliance. We merely intend to demonstrate that, given the reality of scientists being judged, rightly or wrongly, using citation metrics, the pagerank-index can return a fairer comparison than other metrics currently in use. Let us also emphasise that Google Scholar is a public database and our analysis in this section is based on the publicly available data in Google Scholar alone, and does not include any contributions from a scientist which may not be in Google Scholar or not attached to the above mentioned profile. Having mentioned these caveats, let us consider Table 1, which shows the top twelve scientists in the field of Quantum Game Theory, according to h-index. Note well that we are using the ‘internal’ h-index, which is computed using only the papers in the field and therefore typically much lower than the ‘overall’ h-index of scientists. We do not want to use the ‘overall’ h-index since that purportedly measures the scientific contributions of authors to all fields, not just quantum game theory. The same table also shows the pagerank-index, and ranking based on pagerank-index, of each of these authors, while Table 2, in contrast shows the top 12 authors in the field with respect to pagerank-index, and the h-index of each of these authors. We can observe that certain authors, such as *A. Iqbal* (h-Rank = 1, π-Rank = 1) and *A.P.Flitney* (h-Rank = 3, π-Rank = 2) maintain their positions at the top of the table with respect to both ranking systems. Other authors, such as *D.Abbott*, leap several places up (12th to 3rd) when pagerank-index is applied. Several other authors, such as *J. Du* (h-Rank = 4, π-Rank = 14), *R. Han* (h-Rank = 7, π-Rank = 46), *X. Xu* (h-Rank = 7, π-Rank = 46), *X. Zhou* (h-Rank = 7, π-Rank = 46) receive significantly lower ranks when the pagerank-index is applied, compared to h-index. Looking at the data available in the Google Scholar Profile, it is very easy to see how and why this happens. Let us take researcher *D. Abbott*, for example. The top papers by *D. Abbott* were published in highly reputable journals, such as Nature, Physical Review Letters, and Statistical Science. Therefore, the chance of his citations coming from other highly reputed journals is high. More importantly, he has only an average 2.8 authors for each of his papers in this field, which means his papers had typically been authored by two or three authors, including himself. These factors would not be taken into account by h-index, which merely counts the number of citations an author receives, but such details are rightly taken into account by pagerank-index, which returns a relatively higher score for authors who put in a higher ‘share’ of individual work into a paper and publish in, and more importantly get cited by, higher quality journals.

**Table 1:** The top 12 scientists as ordered by their h-index in the field of quantum game theory.

Author	h-index	h-percentile	pagerank-index	Papers	Avg. Authors per Paper	rank-h	rank-π
A Iqbal	11	99.7	99.8	49	2.65	1	1
AH Toor	11	99.7	98.8	20	2.1	1	9
AP Flitney	10	99.6	99.7	26	2.34	3	2
J Du	9	99.2	98.6	15	4.66	4	11
H Li	9	99.2	97.5	12	4.66	4	19
J Sladkowski	9	99.2	98.9	23	2.26	4	8
NF Johnson	8	98.6	98.2	13	2.076	7	14
X Xu	8	98.6	95.1	8	5.5	7	38
X Zhou	8	98.6	92.1	9	5.33	7	62
R Han	8	98.6	79.59	8	5.5	7	160
EW Piotrowski	8	98.6	99.4	22	2.22	7	4
D Abbott	7	98.4	99.6	37	2.81	12	3

**Table 2:** The top 12 scientists as ordered by their pagerank-index in the field of quantum game theory.

Author	h-index	h-percentile	pagerank-index	Papers	Avg. Authors per Paper	rank-h	rank-π
A Iqbal	11	99.7	99.8	49	2.65	1	1
AP Flitney	10	99.6	99.7	26	2.34	3	2
D Abbott	7	98.4	99.6	37	2.81	12	3
EW Piotrowski	8	98.6	99.4	22	2.22	7	4
A Nawaz	4	96.6	99.3	15	1.86	21	5
DA Meyer	5	97.4	99.2	9	1.77	18	6
M Ramzan	4	96.6	99.1	14	2.42	21	7
J Sladkowski	9	99.2	98.9	23	2.26	4	8
AH Toor	11	99.7	98.8	20	2.1	1	9
T Cheon	6	97.8	98.7	16	1.93	13	10
J Du	9	99.2	98.6	15	4.66	4	11
P Frackiewicz	1	55.5	98.4	6	1.33	87	12

On the other hand, there are certain authors whose rankings go down when pagerank-index is applied. For example, the author *R. Han* drops from 7th to 160th when pagerank-index is applied instead of h-index. A quick perusal in Google Scholar reveals that this author, while also publishing in high impact journals such as Physical Review Letters, often appears to be part of a large group of co-authors. The average number of authors per paper for this author is 5.5 within Quantum Game Theory field as defined above, which means the author has co-authored with typically five or six authors, which is very significantly higher than authors like *D. Abbott*. Furthermore, this author appears as the last author in their most highly cited papers. In fact, the authors *J.Du* (4.7), *X. Xu* (5.5), *X. Zhou* (5.3), and *R. Han* (5.5) all appear to have co-authored several papers together, as part of a relatively large group of authors, as the average number of authors in their papers given in brackets above indicate. Some of these papers have been highly cited. While this clearly gives them all high h-index values, the pagerank-index considers the fact that the co-author pool is relatively large and thus does not give them very high scores.

**Fig 15:**
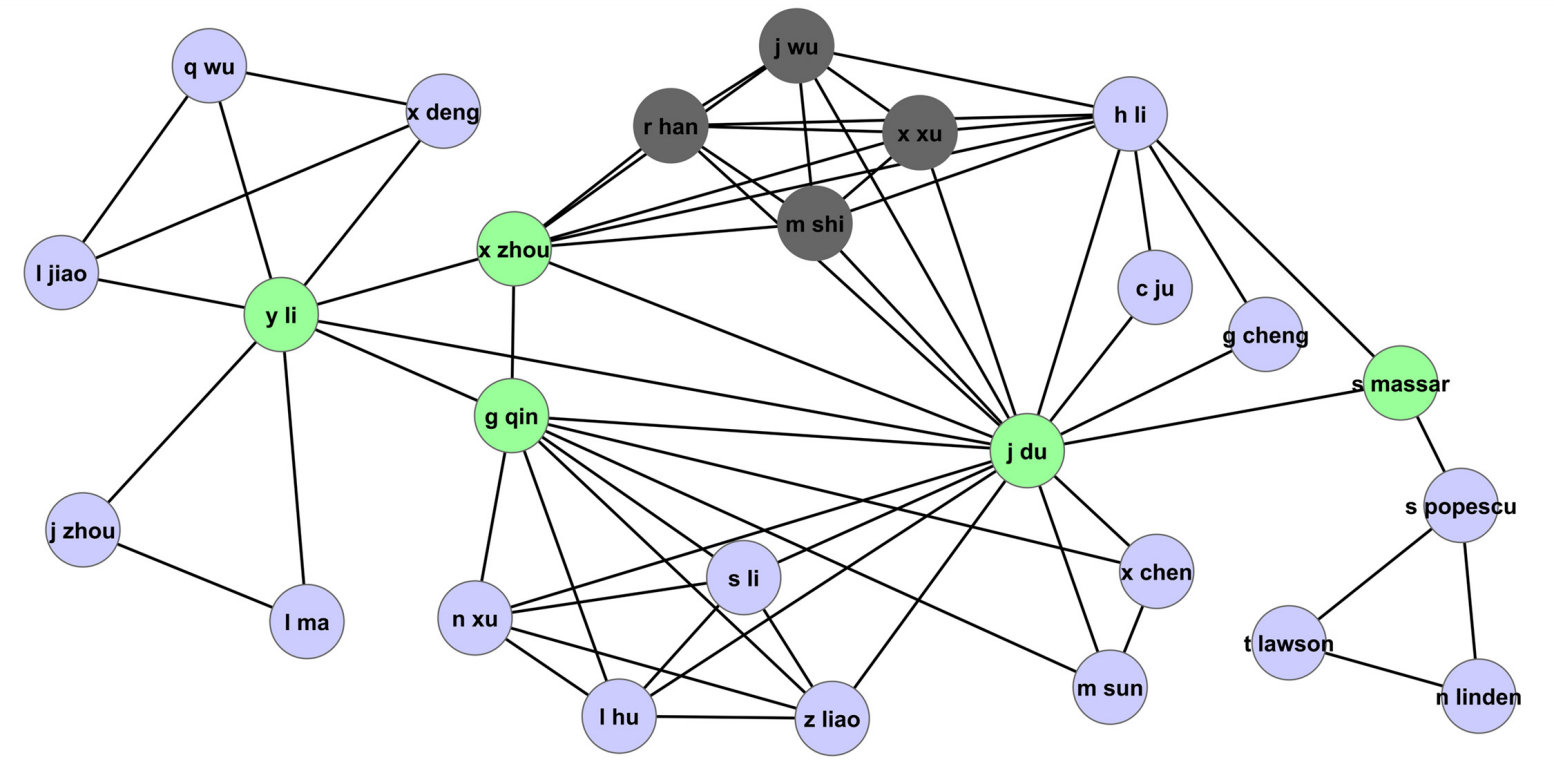
Part of the collaboration network highlighting authors *R. Han* (h-index: 8, pagerank-index: 79.6%), *X. Xu* (h-index: 8, pagerank-index: 95.1%), *J. Wu* (h-index: 1, pagerank-index: 89.3%), and *M. Shi* (h-index: 3, pagerank-index: 79.1%) in the field of Quantum Game Theory.

**Fig 16:**
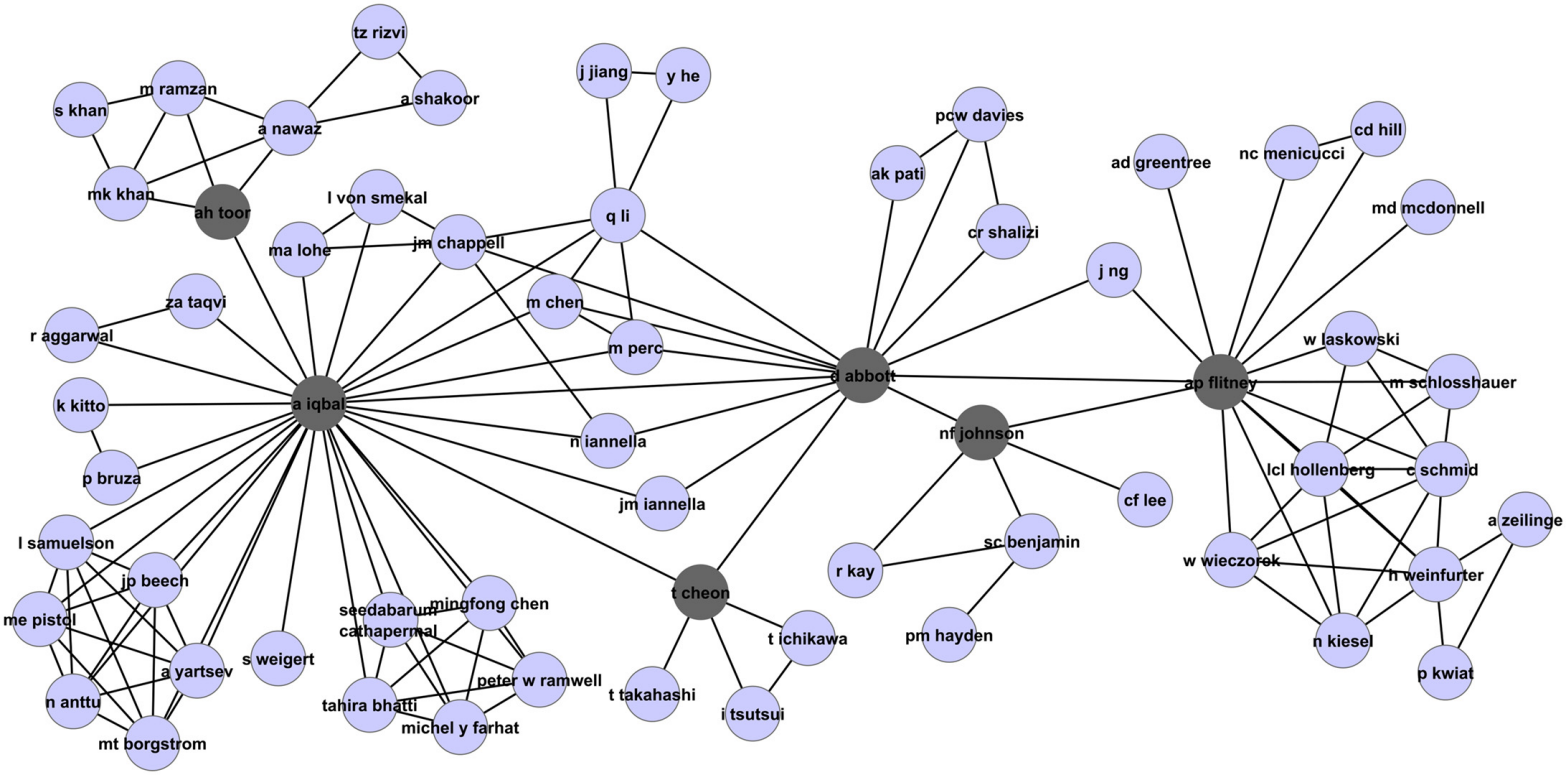
Part of the collaboration network highlighting the prominent authors from the Quantum Game Theory Google Scholar profile. The details of the highlighted authors are listed in Table 2.

**Fig 17:**
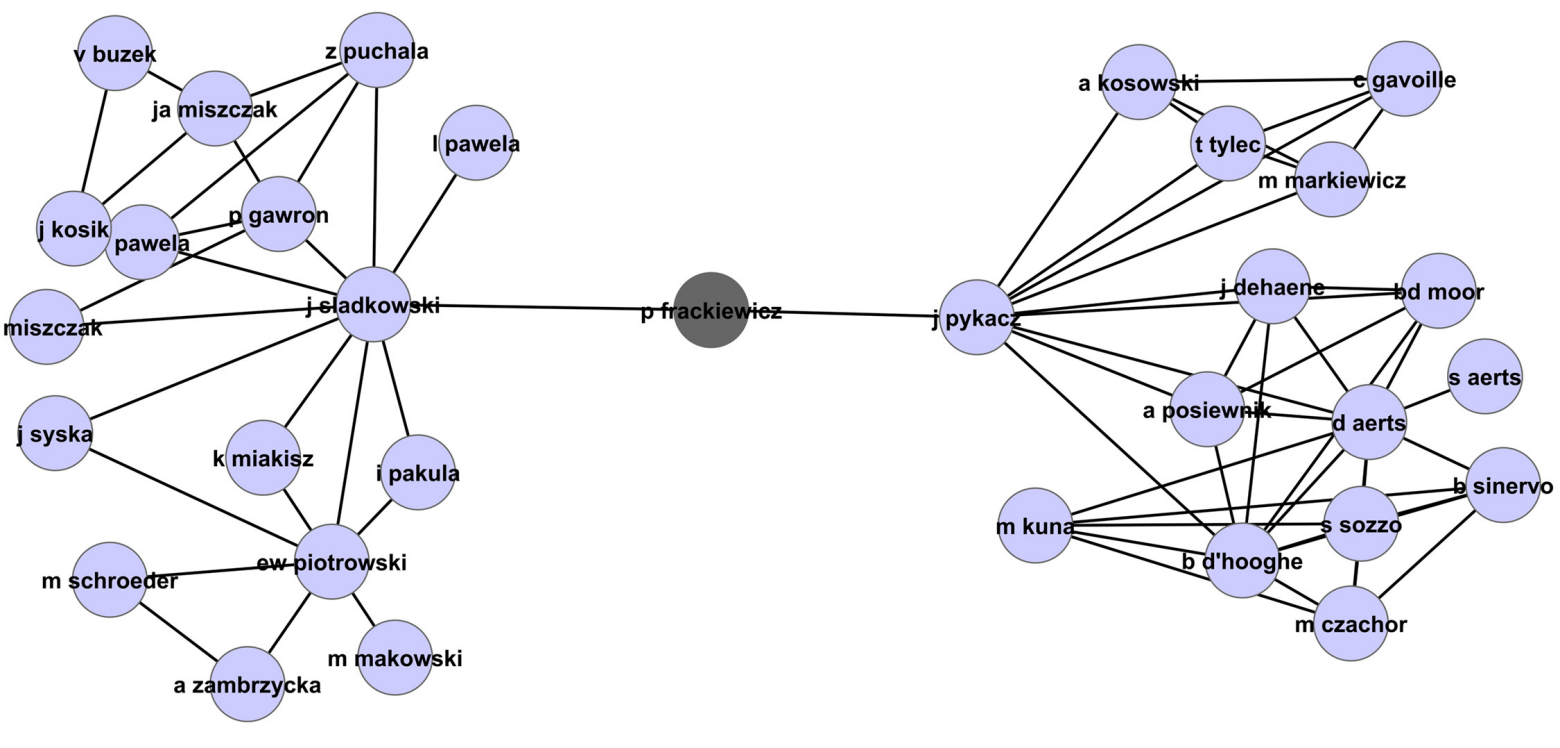
Part of the collaboration network highlighting P. Frackiewics (h-index:1 (h-index percentile 55.5%),pagerank-index:98.4%). It is clear that this author plays an important role in the field by being the ‘bridge’ between two sets of authors who work perhaps in two sub-fields. Note that though the collaboration network is not an input in computing the pagerank-index, the pagerank-index is able to recognize and reward authors who perform such an important role in the development of the field, as indicated by the relatively high pagerank-index of this author. The h-index, being a relatively simplistic citation count measure, fails to recognize this fact.

The collaboration network in Fig 15 lends further credence to our arguments. As highlighted in the figure, it indicates that the author *R. Han*, for example, has only six co-authors in this field, and these authors seem to be in a nearly fully connected subnetwork, meaning they wrote most of their papers in this field as a group. Particularly, authors *R. Han, X. Xu, J. Wu*, and *M. Shi* seem to have written no paper together with authors outside this subnetwork. This subnetwork has co-authored together some highly cited papers, so it is not surprising that members of this subgroup, and particularly the authors mentioned above, have their ranks within the field change considerably when pagerank-index is used (*X. Xu*: h-Rank = 7, π-Rank = 46, *R. Han*, h-Rank = 7, π-Rank = 46, *M. Shi*, h-Rank = 27, π-Rank = 147). Author *J. Wu* who only has three papers and an h-Rank of 87, however, does not seem to be affected that much. On the other hand, author *D. Abbott* as highlighted is not part of a ‘dense’ subnetwork shown in Fig 16, but it is evident that he has high ‘centrality’ within the co-authorship network, which means his contributions help form the ‘backbone’ of the field and he has helped expand the field in various directions, which justifies his ‘jump’ from 12th to 3rd when pagerank-index is used. Another author who has obviously high centrality within the author network, performing the function of bridge between two group of authors (even though he has only two co-authors, one each from each of these groups) is *P. Frackiewics* as shown in the Fig 17. This author significantly gains in rank when pagerank-index is used (h-rank = 87, π-Rank = 13). Such a jump is justified by his definitive contribution to the field. Therefore, we argue that the pagerank-index is a lot fairer in ‘teasing out’ highly impactful contributors in the field, since it (directly or indirectly) considers subtle factors that the h-index cannot consider, including number of co-authors, ‘centrality’ and ‘visibility’ in the field etc. This is all the more interesting because the pagerank-index does not use the *co-authorship (collaboration)* networks in its calculations at all—it only uses the citation networks. However, due to its in-built fairness and emphasis on subtle nuances, the authors who make definitive contributions by being the backbone of genuine collaborations are rewarded. Having said that, it should be mentioned very clearly that we do not presume to criticise the contribution of the authors whose π-Rank is lower than their h-rank. It is perfectly legitimate to work in large groups, and there could be scientific reasons for working in such groups. It is not a weakness of the authors concerned, but a weakness of the h-index, that authors working in such large groups gain disproportionally higher visibility. We emphasise that the pagerank-index is a better metric because it is able to go beyond mere citation counts and rewards definitive contributions of authors in many ways, as described above.

**Fig 18:**
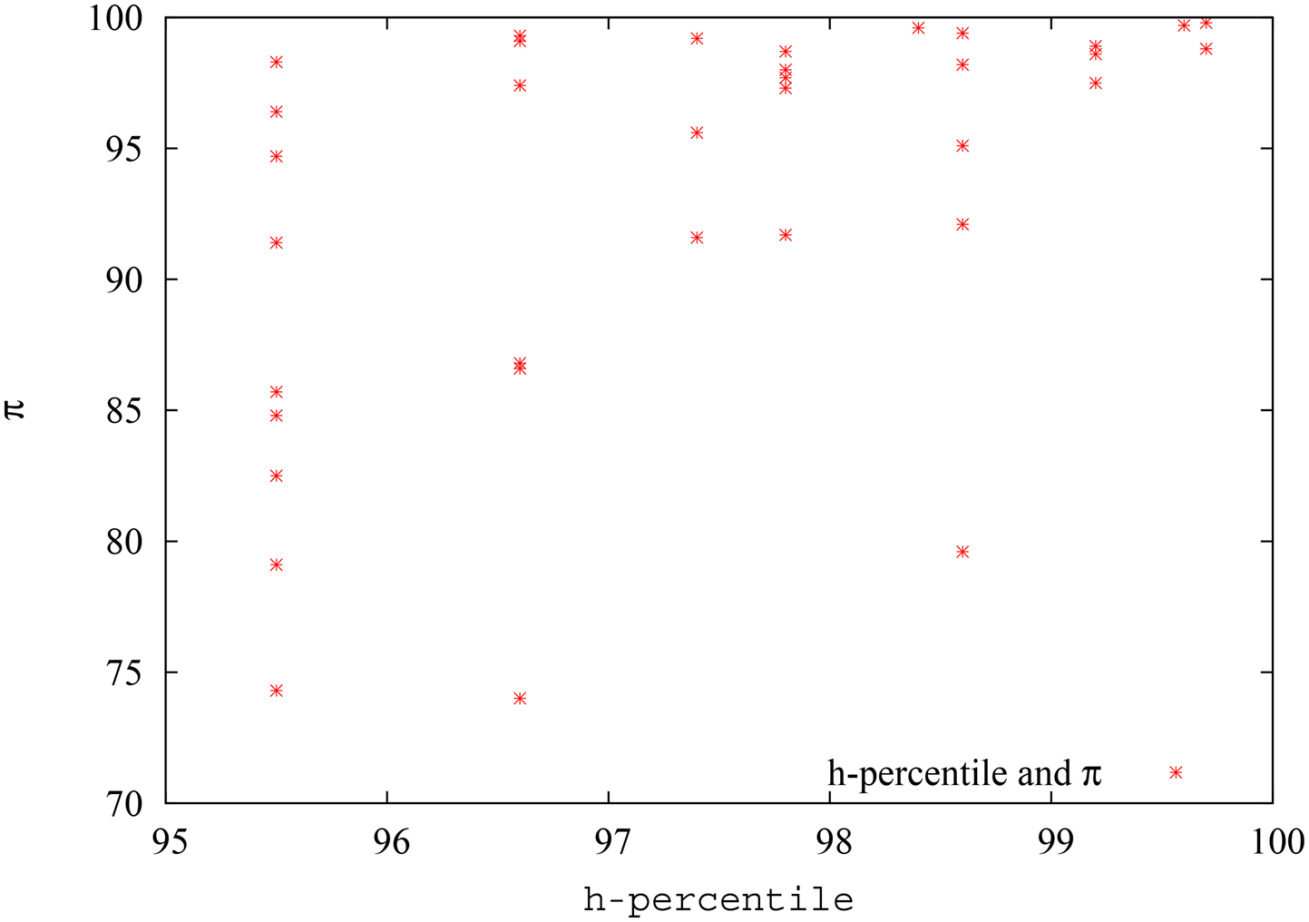
The h-index and pagerank-index of the best 5% authors (according to h-index) in the field of quantum game theory. Since the pagerank-index is a percentile, percentile values were used for the h-index as well, rather than actual h-index values. Note here that the pagerank-index value varies from 70% to 100%. That is, some authors who are among the top 5% in terms of h-index are not even among the top 25% when pagerank-index is considered.

In Fig 18, we show the pagerank-index vs percentile (internal) h-index plot for the field of Quantum Game Theory as defined in Google scholar. We convert the h-index also to percentile, since it makes it easier to compare the two metrics this way, and also because otherwise it might be argued that the variation is due to a percentile being compared with a ‘direct’ score. We avoid showing the h-index percentile range below 95%, since h-index is a discrete quantity and there are only two data points below 95%, corresponding to h-index = 1 (88.9%) and h-index = 0 (55.4%), and showing these would reduce clarity of the plot. We can see from the figure that there is a lot of variation for pagerank-index value for each percentile h-index points. Some authors who score 95.4% h-percentile, score as low as 74% in terms of pagerank-index, while others score as high as 98%. It could be demonstrated, by individual analysis as has been done in the previous paragraphs, that those authors who have low pagerank-index value either have typically co-authored with a larger pool of authors, or have received most of their citations from low-impact papers, while those authors who score highly have relatively worked with a smaller pool of authors and received their citations from more reputable sources, thus justifying their high pagerank-index. Similarly, among authors who score 96.5% h-percentile, there is a range of pagerank-index values from 74% to 99%. The three data points at the right top extreme represent *A. Iqbal, A.H.Toor* and *A.P. Flitney*, who have scored well both in terms of h-index and pagerank-index as discussed above. This plot again shows that pagerank-index can be used to ‘tease out’ different levels of contributions from authors who are all at the same level when h-index is used, because h-index just uses citation count while pagerank-index is more nuanced.

#### Grouping authors based on publication tendencies in Quantum Game Theory field

In the previous subsection, we considered individual authors to show how some authors whose definitive contributions are obfuscated by h-index come to prominence when pagerank-index is used for comparison. We could also demonstrate this by dividing authors into two ‘groups’, as has been done in the simulation experiment. However, since we do not know about the motivations behind real-world authors in choosing certain patterns of interactions, we could divide authors only based on data about their papers. Nevertheless, to generalise the findings above, let us extract a group of ‘collaborative’ authors and a group of ‘non-collaborative’ authors from the quantum game theory field. We define that authors who had, on average, more than 3.5 co-authors per paper are collaborative, while those who had less than 2.0 co-authors per paper are non-collaborative. For clear separation, we do not include authors who are in between these bounds into either group. To avoid anomalies, we only consider those authors who published more than 5 papers and been cited more than 5 times.

**Fig 19:**
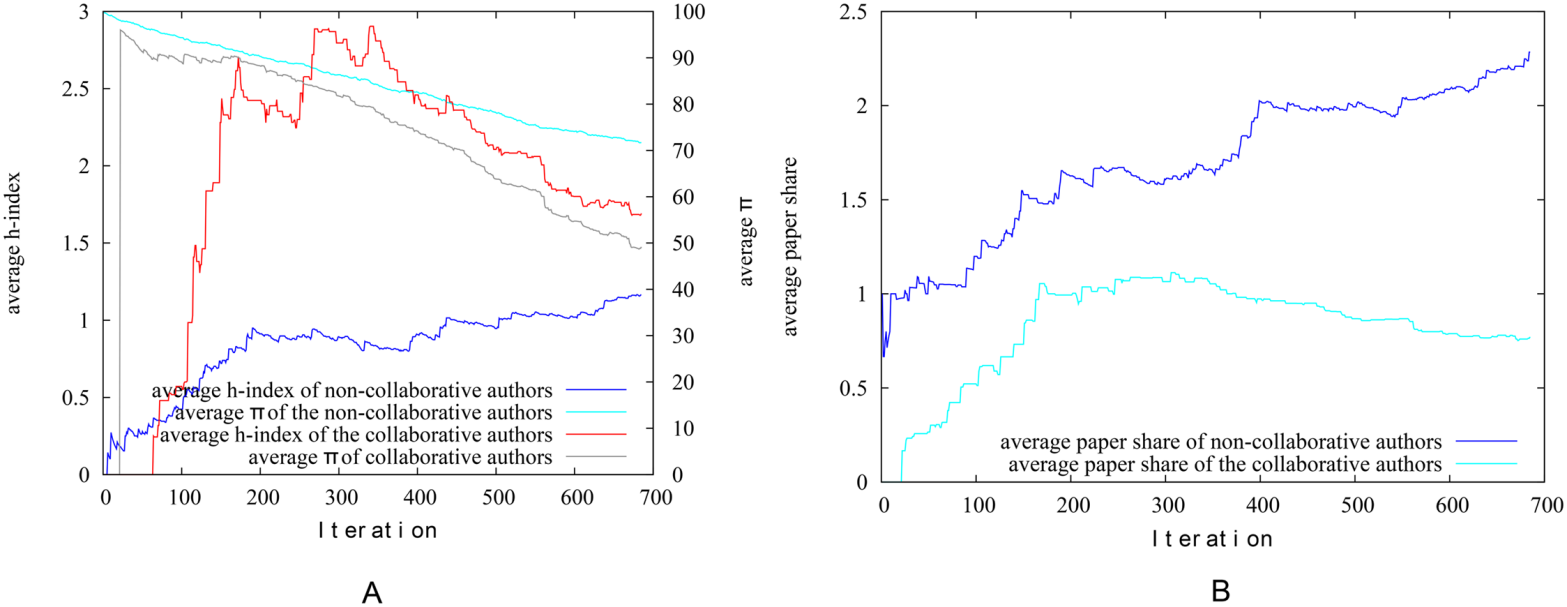
(A) The variation of h-index and pagerank-index for two groups of authors during the evolution of quantum game theory field. The x-axis corresponds to each new paper added and the time line of the evolution is from 1955 to 2014. One group of authors are classified as ‘collaborative’ and another group as ‘non-collaborative’. The way this classification was done is explained in the text. It is clear that while the h-index favours the ‘collaborative’ authors, the pagerank-index, in general, tends to favour the ‘non-collaborative’ authors. (B) The average ‘papershare’ of collaborative and non-collaborative authors during the evolution of quantum game theory field. The ‘papershare’ is calculated as the summation of proportional contributions made to papers. For example, if an author has contributed two papers each with two other co-authors, he has a total of 4/3 paper-shares. It is clear that the ‘non-collaborative’ authors work harder and have more ‘paper-shares’ than collaborative authors. Contrasting with part (A), we may see that the pagerank-index highlights this fact by favouring the ‘non-collaborative’ authors, while the h-index arguably unfairly favours collaborative authors who on average produce less ‘paper-shares’.

Fig 19(a) presents the average h-index and pagerank-index of collaborative and non-collaborative authors over time, while Fig 19(b) presents the average ‘paper-share’ of authors over time. The ‘paper-share’ is simply the total of proportions of papers an author has written. For example, if an author had written two papers and in each of these there were two co-authors, this author has in total 2/3 ‘paper-shares’: it is as if this author wrote 2/3 of a paper by himself. We may notice that there is a clear propensity for the collaborative authors to score a higher h-index while it is not possible if pagerank-index is used. In fact, the pagerank-index is on average higher for non-collaborative authors. This however is justified when we look at Fig 19(b), since we could see that the non-collaborative authors have in fact worked harder and contributed more ‘paper shares’ in this dataset over time. The pagerank-index on average reflexes this, while the h-index is in fact higher for collaborative authors on average! This result confirms the observations made previously that authors who collaborate more are, perhaps unfairly, given an advantage by h-index, which pagerank-index nullifies. Similar experiments with this dataset could be conducted for other scenarios (1 and 3) described in the simulation experiments above as well. However, since we have no evidence to suggest that some authors in this particular field deliberately publish documents to self-cite, and to avoid belabouring the point unnecessarily, we avoid showing results for such groupings in this paper.

#### Correlation between h-index and pagerank-index

We have emphasized that the pagerank-index is fairer than h-index in many scenarios, and highlights the contributions of the authors who are obfuscated by the h-index. Yet, it is a measure based on citations also. How much correlation does it have with h-index in a real community of authors? To see this, we ranked authors in the field of Quantum Game Theory (as defined by the dedicated Google profile) using both pagerank-index and h-index, and measured the correlation. The results of this experiment are given as a plot in S1 Appendix. The important result from this experiment is that the Pearson’s correlation between the h-ranks and the p-ranks has a value of 0.489. This signifies that the two systems are closely correlated, yet the pagerank-index is a metric sufficiently different from h-index to have value of its own.

### Demonstrating the utility of pagerank-index in HEP-Theory dataset

Although the Quantum Game Theory data set enables us to observe the evolution of a citation network and how pagerank-index and h-index can evolve with the citation network, it is a relatively small network with 685 publications and 785 authors. As such, the second citation network we examine is the High Energy Physics (HEP-TH) dataset as provided by Leskovec et al. [17]. It is a static snapshot of the HEP-TH citation network as at April, 2003 featuring 29555 publications over the duration of January, 1993 to April, 2003. There are 15332 authors altogether in this dataset and it has been curated from Arxiv as a part of the KDD cup 2003 [26]. Unlike the Quantum Game Theory data set, though, we cannot look at the ‘evolution’ of a particular field from the first paper to its present position: that is the disadvantage in using such a large data set.

We used the dataset to construct the citation network using a computer program that used the provided text data to map nodes and edges of the citation network. We then used the provided meta data in order to build the collaboration network for supplementary analysis, though, as mentioned, the pagerank-index only uses the citation network in its computation. The IDs of the papers and authors correspond to the time of their entry to the dataset, and again only ‘internal’ citations were considered(Network level characteristics of this dataset are listed and the degree distribution of the citation network is presented in S1 Supplementary Material).

We first present some qualitative observations to illustrate the utility of pagerank-index in this data set. Hence let us consider the Table 3 presenting the top twelve scientists of the HEP-TH dataset as ranked by h-index. Contrast this to Table 4 which presents the top twelve scientists of the HEP-TH dataset as ranked by pagerank-index. We can observe that some authors like *A.A Tseytlin* manage to keep their position intact (p-rank = 3, h-rank = 3). Some authors who are below the top twelve in Table 3 come to prominence when ranked using pagerank-index. For example, *Shin’ichi Nojiri* was below the top twelve if ranked using h-index but is in the 4th position when ranked using pagerank-index. Likewise, *E. Elizalde* is ranked 229th using h-index but leaps to the 6th position when pagerank-index is used. These authors have published in high impact journals like *Science* which pagerank-index implicitly takes in to consideration (because many papers citing their papers also would have appeared in *Science*, cited by many others) while h-index does not. As opposed to this, authors like *C.N Pope* and *P.K Townsend* are ranked highly when h-index is used (6 and 10 respectively) but their ranking goes down significantly when pagerank-index is used (25 and 47 respectively). This is mainly because both of these authors have a relatively high average authors per paper value and they have been second / third authors more often, which factors the pagerank-index takes into consideration but the h-index does not.

**Table 3:** The top 12 scientists as ordered by their h-index in the HEP-TH dataset.

Author	h-index	h-percentile	pagerank-index	Papers	Avg. authors per paper	rank-h	rank-p
Edward Witten	54	99.9	99.9869	94	1.5744	1	2
Ashoke Sen	47	99.9	99.9934	89	1.5505	2	1
A.A. Tseytlin	41	99.9	99.9804	109	1.7155	3	3
Cumrun Vafa	40	99.9	99.8760	77	2.0389	4	19
Michael R. Douglas	35	99.9	99.8695	52	2	5	20
H. Lu	32	99.9	99.9543	123	3.5284	6	7
C.N. Pope	32	99.9	99.8369	128	3.4843	6	25
Nathan Seiberg	32	99.9	99.5238	42	2.1666	6	73
Andrew Strominger	31	99.9	99.7586	56	2.1607	9	37
Joseph Polchinski	29	99.9	99.7912	45	1.8222	10	32
P.K. Townsend	29	99.9	99.6934	55	2.3818	10	47
Gregory Moore	29	99.9	99.6282	53	2.3396	10	57

**Table 4:** The top 12 scientists as ordered by their pagerank-index in the HEP-TH dataset.

Author	h-index	h-percentile	pagerank-index	Papers	Avg. authors per paper	rank-h	rank-p
Ashoke Sen	47	99.9	99.9934	89	1.5505	2	1
Edward Witten	54	99.9	99.9869	94	1.5744	1	2
A.A. Tseytlin	41	99.9	99.9804	109	1.7155	3	3
Shin’ichi Nojiri	20	99.7	99.9739	94	2.4574	29	4
Nathan Berkovits	23	99.8	99.9673	59	1.3898	20	5
E. Elizalde	11	98.1	99.9608	77	2.5064	229	6
H. Lu	32	99.9	99.9543	123	3.5284	6	7
Zurab Kakushadze	21	99.8	99.9478	63	1.7777	25	8
Donam Youm	18	99.5	99.9412	55	1.2909	53	9
Sergei V. Ketov	10	97.5	99.9347	46	1.2173	291	10
Itzhak Bars	18	99.5	99.9217	44	1.25	53	11
Valeri V. Dvoeglazov	7	94.8	99.9217	41	1.0487	606	11

**Fig 20:**
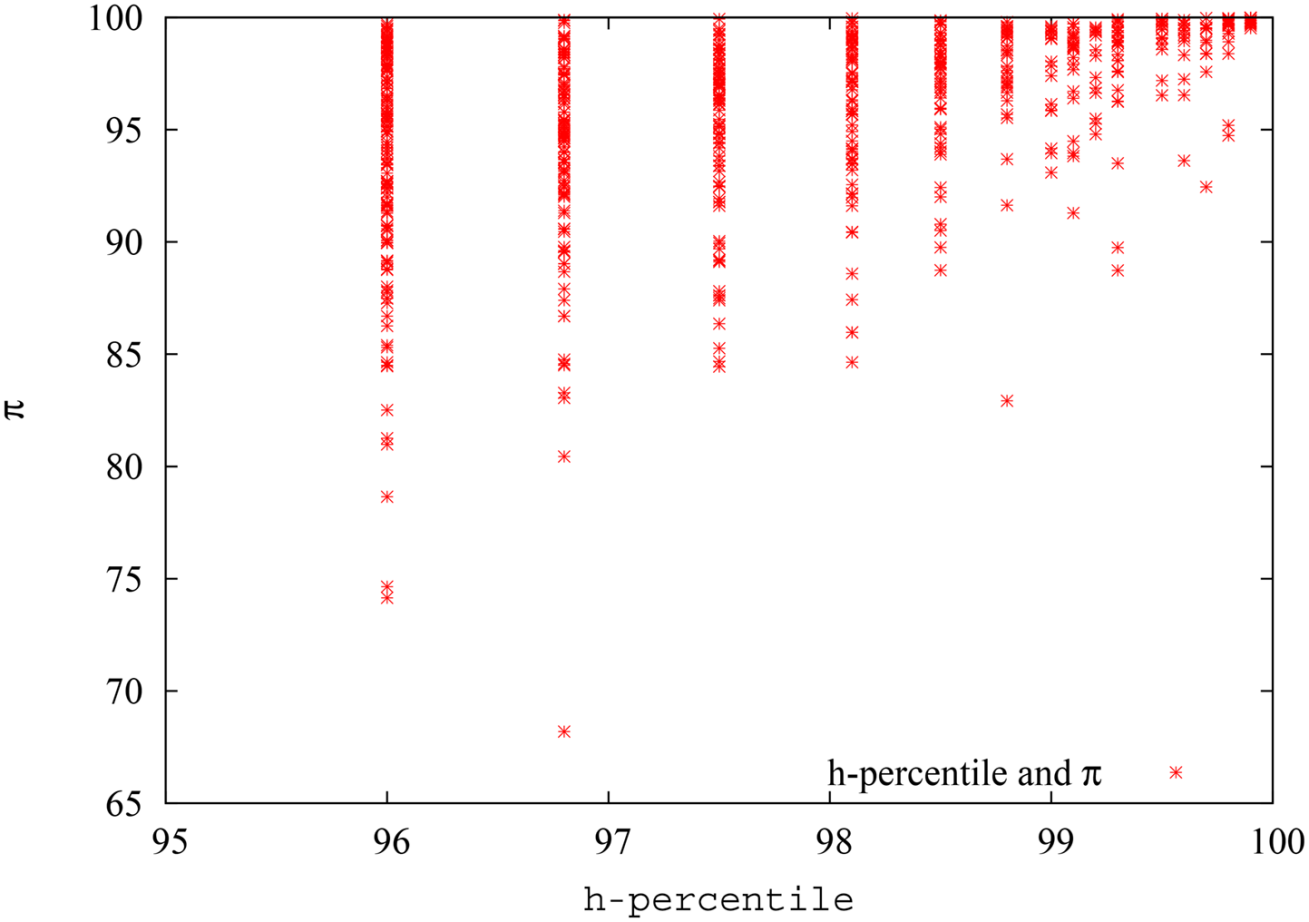
The h-index and pagerank-index of the best 5% authors (in terms of h-index) in the HEP-TH dataset. Since the pagerank-index is a percentile, percentile values were used for the h-index as well, rather than actual h-index values. Note here that the pagerank-index value varies from 65% to 100%. That is, some authors who are among the top 5% in terms of h-index are not even among the top 25% when pagerank-index is considered.

In Fig 20 we present the plot of pagerank-index vs h-index for this data set. In order to compare pagerank-index directly with h-index, we convert h-index values to a percentile and use the h-percentile in the plot, as before. In this data set, the Pearson correlation between the two ranking systems is 0.59. Again, only the top 5% of the h-index values (95% and above) are shown in the figure for the purpose of clarity and as can be seen, the corresponding pagerank-index values have a range from 65%—100%. This implies that some scientists who are in the top 5% of the h-index may not even make it to the top 25% if ranked using pagerank-index. An author who scores 99% using h-index may score as much as 99.9% and as less as 91.8% if pagerank-index is used. These variations of the authors can be individually explained as before, using various factors like their percentage of self-citations, their level of collaboration, the impact of their publications and the centrality of their positions in the corresponding collaboration network. By detailed analysis we can observe that the authors who tend to co-author with a small pool of authors, publish in high impact platforms, and have a high centrality in terms of collaboration may score higher than others when ranked using pagerank-index and conversely, authors who tend to significantly self-cite, co-author papers with a large pool of authors and publish in low impact forums may score lower when ranked using pagerank-index. The top right end of the same figure represents the scientists who are ranked at the top in both h-index and pagerank-index. A few examples would be *Ashoke Sen*, *Edward Witten* and *A.A. Tseytlin* who are all prominent and well respected scientists in the field of theoretical high energy physics. Despite such exceptions, it is clear from analysing this data set that the pagerank-index is again a more nuanced index, and provides for a deeper understanding of the relative productivity and contribution of scientists compared to the existing measures.

## Discussion and Conclusions

Even though there is significant and justified criticism against it, measuring and comparing the scientific output of researchers in terms of their publications and citations has become an important exercise within scientific community, for purposes of employment, funding, awards and the like. Therefore it is of importance to continually analyse and further develop methods and metrics which are used for this purpose. At present, perhaps the most famous and well utilized metric in this regard is the h-index. Even though many shortcomings in this metric have been pointed out, the proposed alternatives have not succeeded in addressing all of them together, and none of them have addressed, in a principled manner, the very important shortcoming that the citations are not weighted. Using impact factors for this purpose again has a number of well-documented shortfalls.

In this paper, we propose a new metric, the pagerank-index, which we contend is a ‘fairer’ metric in a number of ways compared to the h-index and other existing measures. The pagerank-index is a dynamic measure computed from the underlying citation network of the author community. It is a principled and nuanced measure, because it makes no assumptions about the status of the journals, conferences or authors other than using the actual citation data, yet considers the infinite levels of feedback a paper receives rather than the simple citation count. It is a sophisticated and elegant measure since its method of computation ensures the ‘position’ of authors within their communities, their relative standing, and their historic contribution in developing a field will be rewarded, without assigning or using crude weights to represent any of these factors.

We have undertaken extensive studies to validate the utility of the new measure. Particularly we used both a simulated system of authors and real-world databases to establish its comparative merits. We have focused primarily on comparing pagerank-index with h-index, since h-index is itself an improvement on other previous measures, as Hirsch explains [2]. We used a realistic simulated system so that we can have the freedom of ‘assigning’ inherent behaviour traits to authors, and establish how these traits influence both the h-index and pagerank-index. We showed that, compared to h-index, the pagerank-index is robust to authors deliberately self-citing or ‘group citing’, including creating sub-standard papers for this express purpose; authors working in large groups which, intentionally or otherwise, helps increase their citation counts in ways disproportionate to their effort; and authors focusing on quantity of papers rather than quality, which, again intentionally or otherwise, helps to increase their citation counts disproportionately. In all these scenarios, we showed that the pagerank-index reduces the advantage authors with a particular ‘trait’ or publication habit have over others who have the opposite trait. We also showed that such reduction of advantage is always very significant, five to ten times at the least. Thus we showed that the pagerank-index is a ‘fairer’ metric which rewards scientists equally who display similar levels of talent and effort, despite having different publication habits.

We then used two data sets to test the new metric in a real world setting: (i) The Quantum Game theory field as defined by the dedicated Google scholar profile, from which we curated the data (ii) The publicly available HEP-Theory data set [17]. Using the first data set, we showed that the pagerank-index highlights contributions of authors which are obscured by the h-index, by considering not only citation count but also the sources from which the citations are received, the author count, the ‘centrality’ of an author in his/her respective field, and how a particular paper of an author aided in later development of the field. Thus, we showed that the pagerank-index is more nuanced and rewards authors who deserve higher recognition. We analysed the HEP-theory data base and made similar observations, despite the fact that this data base is only a ‘snapshot’ of the relevant field (high energy physics) and does not constitute information from the inception of the field, and in any case does not include all papers in the HEP-theory field. Therefore, despite the limitations in ‘defining’ a field, both studies showed how the pagerank-index could highlight information which could be obscured by traditional measures. Importantly, we showed that the correlation between pagerank-index and h-index is about 50% generally (0.49 for Quantum Game Theory and 0.59 for HEP-theory). A non-correlation or negative correlation would have meant the pagerank-index measure departs too far from the established h-index, causing difficulties in its uptake. A near complete correlation would have meant the pagerank-index does not bring a lot of additional value. However, a 50% correlation means the pagerank-index is very valuable, as it has sufficient overlap with other citation-based measures yet brings in considerable new information, particularly regarding understanding who is at the ‘top’ of the field.

The pagerank-index has other advantages that traditional measures do not. If a scientist dies or stops working in the field, their h-index would not increase further beyond a limit, since it is limited by the number of papers. Neither would it decrease. However, there is no limit to how much their pagerank-index can increase, and it can indeed decrease, reflecting their real ‘relevance’ to the field at that point in time. For example, if a scientist writes a single masterpiece which kicks off a particular field and then dies, she will have only an h-index of one no matter how highly cited, and may not even have that many citations. However, she will have a very high pagerank-index, even if she gets cited only a few times but those papers which cite her work go on to be cited thousands of times, and her pagerank-index will keep increasing as long as papers which cite her work get cited and so on. The pagerank-index also has the advantage that it can be applied in many levels of abstraction: for a particular field, a country, a community of authors, as we have demonstrated in this paper, or to scientific community as a whole.

A few comments are warranted to contrast the new index with grading systems used in commercial tools such as *Researchgate*. In recent times, online social networking tools, such as Researchgate, have introduced scores for scientists which are supposed to quantify their scientific excellence. However, a detailed look at their scoring methods reveal that they are far from justified: in fact, they are worse than h-index or number of citations in terms of being objective measures of scientific performance. For example, Researchgate states, quoted verbatim [27], that “Once you’ve created content, your score is calculated based on how other researchers interact with your work, how often, and who they are. The higher their score, the more yours will increase.” While Researchgate has not revealed the exact algorithm behind the RG score, it seems that those scientists who do more online activity within Researchgate are rewarded: for posting, for answering questions, for ‘following’ and ‘being followed’, for sharing data etc. Therefore, the RG score simply serves the purpose of making people spend more time within the tool. While the pagerank-index also will need to be implemented in commercial platforms for wider uptake, it does not require a scientist to spend time or even be a member of any online forum, database or tool: simply publishing publicly accessible and indexable research papers will suffice. In any case, any tool which uses an algorithm not publicly available to be scrutinised will not be relied upon by scientific agencies for comparison of researchers.

The pagerank-index is no silver bullet though and has some shortcomings. It is not a measure a scientist can compute for herself, but needs to be implemented in a large scholarly data base. However, this is not a major weakness since those who need to use pagerank-index for funding and employment purposes will not rely on scientists to provide their own pagerank-index, but will look up scholarly data bases to find this information about a particular scientist. Of course, the calculation is limited by what papers are actually in the data base, however this is the same scenario with h-index anyway and data bases such as Google Scholar are very nearly taken as sacrosanct. Another disadvantage is that it is computationally intensive, yet again it is not prohibitively so. In any case, Google uses pagerank for searching on a network (World Wide Web) which is presumably many orders of magnitude larger than any citation network of academic documents. Yet since Google searches are fast and accurate enough to be relied upon by millions, the pagerank-index can also be calculated quickly and accurately, if properly implemented in a scholarly database. It also could be noted that pagerank-index suffers from the fact that pagerank algorithm itself could be ‘massaged’. For example, Zhang et al. [28] observes that pagerank could be vulnerable to ‘collusion’, loosely defined as ‘the manipulation of link structure by a group of nodes’. Nevertheless, it is obvious that such manipulation is considerably more involved than simply citing each other, and while it could be done in the World Wide Web by information technology experts, the virtual citation network is less amenable for such complex manipulation. In any case, the modifications to pagerank proposed by [28] could be applied equally well in pagerank-index calculation if needed, to minimise the impact of ‘collusion’. The final and obvious weakness is that it is still a citation based measure, and would not favour scientists whose brilliant work for some reason is not noticed, however this weakness is very hard to overcome by any quantitative approach.

It is our hope that the pagerank-index will be extensively used by scientific community in quantifying the scientific output of researchers.

## Supporting Information

S1 Appendix (PDF)

S1 Supplementary Material (PDF)

## References

[1] Kozak M, Bornmann L (2012) A new family of cumulative indexes for measuring scientific performance. PloS one 7: e47679 10.1371/journal.pone.0047679 23118890 PMC3485265

[2] Hirsch JE (2005) An index to quantify an individual’s scientific research output. Proceedings of the National Academy of Sciences of the United States of America 102: 16569–16572. 10.1073/pnas.0507655102 16275915 PMC1283832

[3] Bornmann L, Daniel HD (2007) What do we know about the h index? Journal of the American Society for Information Science and Technology 58: 1381–1385. 10.1002/asi.20609

[4] Bornmann L, Mutz R, Daniel HD (2008) Are there better indices for evaluation purposes than the h-index? A comparison of nine different variants of the h-index using data from biomedicine. Journal of the American Society for Information Science and Technology 59: 830–837. 10.1002/asi.20806

[5] Costas R, Bordons M (2007) The h-index: Advantages, limitations and its relation with other bibliometric indicators at the micro level. The Hirsch Index 1: 193–203.

[6] Egghe L (2006) Theory and practise of the g-index. Scientometrics 69: 131–152. 10.1007/s11192-006-0144-7

[7] Konstantin K (2008) Self-citation can inflate h -index. Scientometrics 77: 373–375. 10.1007/s11192-006-1716-2

[8] Jin B, Liang L, Rousseau R, Egghe L (2007) The R- and AR-indices: Complementing the h-index. Chinese Science Bulletin 52: 855–863. 10.1007/s11434-007-0145-9

[9] Van Raan A (2006) Comparison of the Hirsch-index with standard bibliometric indicators and with peer judgment for 147 chemistry research groups. Scientometrics 67: 491–502. 10.1556/Scient.67.2006.3.10

[10] Waltman L, Costas R, Jan van Eck N (2012) Some Limitations of the H Index: A Commentary on Ruscio and Colleagues’ Analysis of Bibliometric Indices. Measurement 10: 172–175.

[11] van Nierop E (2009) Why do statistics journals have low impact factors? Statistica Neerlandica 63: 52–62. 10.1111/j.1467-9574.2008.00408.x

[12] Page L, Brin S, Motwani R, Winograd T (1998) The PageRank Citation Ranking: Bringing Order to the Web. Technical report. doi: citeulike-article-id:3283 URL http://citeseerx.ist.psu.edu/viewdoc/summary?doi=10.1.1.31.1768

[13] Chen P, Xie H, Maslov S, Redner S (2007) Finding Scientific gems with Google’s PageRank algorithm. Journal of Informetrics. 10.1016/j.joi.2006.06.001

[14] Walker D, Xie H, Yan K, Maslov S (2007) Ranking Scientific Publications using a model of network traffic. Journal of Statistical Mechanics. 10.1088/1742-5468/2007/06/P06010

[15] Ma N, Guan J, Zhao Y (2008) Bringing pagerank to the citation analysis. Information Processing and Managing 44: 800–810. 10.1016/j.ipm.2007.06.006

[16] Pinski G, Narin F (1976) Citation influence for journal aggregates of scientific publications: Theory, with application to the literature of physics. Information Processing and Management 12: 297–312. 10.1016/0306-4573(76)90048-0

[17] Leskovec J, Kleinberg J, Faloutsos C (2005) Graphs over time: Densification laws, shrinking diameters and possible explanations In: Proceedings of the Eleventh ACM SIGKDD International Conference on Knowledge Discovery in Data Mining. New York, NY, USA: ACM, KDD’05, pp. 177–187. 10.1145/1081870.1081893 URL http://doi.acm.org/10.1145/1081870.1081893

[18] Chai J, Hua P, Rousseau R, Wan J (2008) The adapted pure h-index. Proceedings of WIS.

[19] Tscharntke T, Hochberg ME, Rand TA, Resh VH, Krauss J (2007) Author sequence and credit for contributions in multiauthored publications. PLoS Biol 5: e18 10.1371/journal.pbio.0050018 17227141 PMC1769438

[20] Waltman L (2012) An empirical analysis of the use of alphabetical authorship in scientific publishing. CoRR abs/1206.4863.

[21] Hu X, Rousseau R, Chen J (2010) In those fields where multiple authorship is the rule, the h-index should be supplemented by role-based h-indices. Journal of Information Science 36: 73–85. 10.1177/0165551509348133

[22] Albert R, Barabási AL (2002) Statistical mechanics of complex networks. Rev Mod Phys 74: 47–97. 10.1103/RevModPhys.74.47

[23] Simon HA (1955) On a class of skew distribution functions Biometrika: 425–440.

[24] Price DDS (1976) A general theory of bibliometric and other cumulative advantage processes. Journal of the American Society for Information Science 27: 292–306. 10.1002/asi.4630270505

[25] (2008). Quantum game theory google scholar page. URL http://scholar.google.com.au/citations?user=wkfPcaQAAAAJ&hl=en

[26] (2003). Kdd cup 2003. http://www.cs.cornell.edu/projects/kddcup/

[27] (2014). Researchgate. https://explore.researchgate.net/

[28] Zhang H, Goel A, Govindan R, Mason K, Van Roy B (2004) Making eigenvector-based reputation systems robust to collusion In: Leonardi S, editor, Algorithms and Models for the Web-Graph, Springer Berlin Heidelberg, volume 3243 of *Lecture Notes in Computer Science*. pp. 92–104. 10.1007/978-3-540-30216-2_8 URL 10.1007/978-3-540-30216-2_8

