# Supplementary material for: The Pagerank-Index: Going beyond Citation Counts in Quantifying Scientific Impact of Researchers
Source: PLoS One. 2015 Aug 19;10(8):e0134794. doi: 10.1371/journal.pone.0134794 (PMC4545754; doi:10.1371/journal.pone.0134794)
Supplement: S1 Appendix — (PDF) [file pone.0134794.s001.pdf]

# The pagerank-index: Going beyond citation counts in quantifying scientific impact of researchers
# Appendix

Upul Senanayake, Mahendra Piraveenan, Albert Zomaya

May 12, 2015

In this appendix, we show some additional information about the simulation and other experiments we have undertaken.

## Attributes of Paper and Author nodes in the paper-generation simulation

| Paper Attribute | Description |
|---|---|
| Paper ID | This attribute is a unique identifier for each paper which would act as an index. |
| Member | Member is a 'generic' parameter which will take different names for different simulations scenarios. It essentially indicates whether a paper belongs to a particular 'group' or not, based on an inherent publication habit of its authors which varies in each simulation scenario. |
| Number of authors | This attributes dictates how many authors are assigned to each paper object. |
| Author list | The author list will contain the list of authors of a paper. |
| Impact Factor | This represents the impact factor of the paper. Note that this attribute is used in *simulating* the author behaviour only. The pagerank-index does not use impact factors in its calculation and when it is applied to real data sets, the impact factors of corresponding journals are not needed. In the simulated system, though, we use the impact factor in order to come up with a weighted preferential attachment process which models citation behaviours. |
| Number of references | This is a total number of references a paper makes (the length of the reference list). |
| Number of internal references | The number of citations a paper makes to other papers within the particular field studied. Thus, this is always less than the total number of references a paper makes. |
| Page rank value | This is the page rank value of a paper node after the page rank algorithm has been executed and a steady-state value has been reached. |
| Citation count | This is the number of citations a paper *receives*. |

Table 1: Attributes of a Paper Node in the simulated system. Note that 'static' attributes are shown in blue and 'dynamic' attributes are shown in black.

| Author Attribute | Description |
|---|---|
| Author ID | This attribute is a unique identifier for each author and would act as an index. |
| Member | Member is a 'generic' parameter which will take different names for different simulations scenarios. It essentially indicates whether an author belongs to a particular 'group' or not, based on an inherent publication habit which varies in each simulation scenario. |
| Paper list | This is a list of papers authored or co-authored by a specific author. |
| Citation count | This attribute represents the number of citations each author has for all of his papers. |
| h-index | This is the h-index of each author. |
| Page rank value | This attribute is to store the page rank value $\Omega$ of the authors in decimal form. |
| pagerank-index | This attribute stores the pagerank-index of authors (in percentiles). |

Table 2: Attributes of an Author Node in the simulated system. Note that 'static' attributes are shown in blue and 'dynamic' attributes are shown in black.

## Evolved Networks

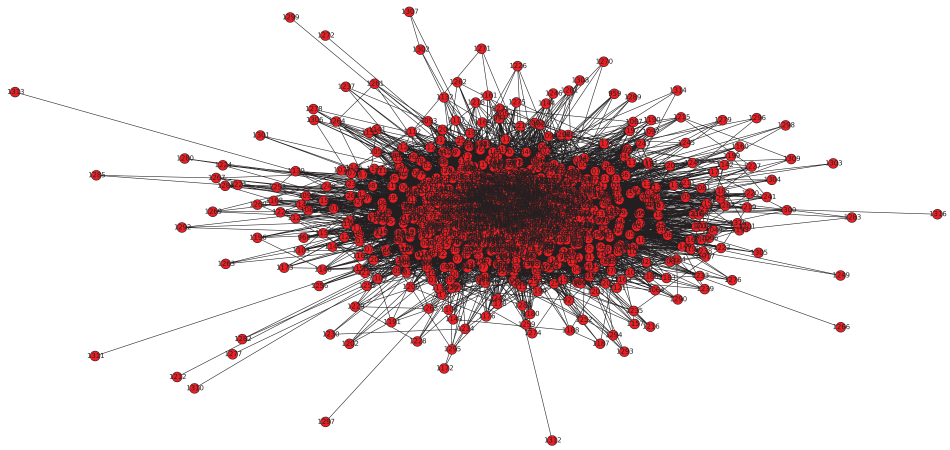

Figure 1: Collaboration network that evolved in a particular run of simulation experiments for scenario 1.

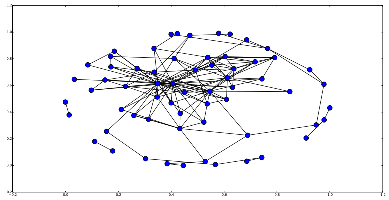

(a) $t = 100$

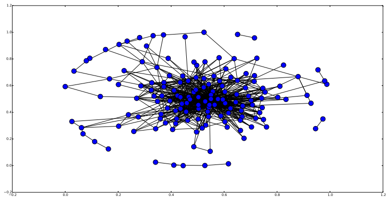

(b) $t = 200$

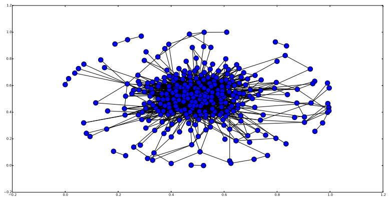

(c) $t = 400$

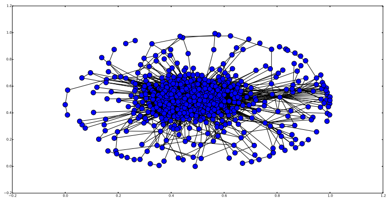

(d) $t = 685$

Figure 2: The citation networks generated from the Quantum Game Theory field (according to Google Scholar) at $t = 100, t = 200, t = 400$ and $t = 685$. The 'timesteps' simply correspond to the number of papers in the field at that time (e.g., at $t = 400$ there were $400$ papers in the field). At the time of access there were 685 papers and 3776 intra-field citations, according to Google Scholar [1].

## Ranks of authors in quantum game theory field using h-index and pagerank-index

We ranked the authors in the quantum game theory field using both h-index and pagerank-index. The ranks are shown in the Fig. 3. As it can be clearly seen from this figure, the rank of some scientists have gone down when ranked using pagerank-index and the ranks of another group of scientists have gone up when ranked using pagerank-index. The ranks that coincides with $y = x$ line represents the ranks of the scientists that has not been changed.

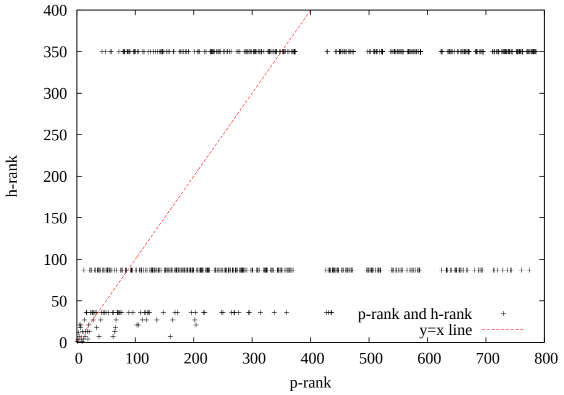


Figure 3: The ranks of the authors as ordered by h-index and pagerank-index from the Quantum Game Theory dataset. The line $y = x$ is also included to better visualize the authors whose ranks are nearly equal when measured by both indices. The range of ranks as ordered by h-index is cut off at 400 because the lowest h-index possible is 0 and the authors who have 0 h-index are ranked at 350 in this particular data set.

## The role of the reset parameter $\alpha$

The reset parameter $\alpha$ in Eq.1 was set at $\alpha = 0.9$ in our experiments. This indicates that the 'random' component is minimal $(0.1 \cdot (1/N))$ compared to the 'endorsement' component $(0.9\cdot$ cumulative endorsement). Therefore, the 'random surfer' behaviour is minimised. Moreover, bearing in mind that pagerank is run multiple times until steady stage is reached, it is easy to see that the influence of 'endorsements' will increase in each iteration and the random component will have very little influence in the final steady state value.

Yet, we consider that it makes sense to have $\alpha < 1$ (a non-zero $(1 - \alpha)$), because there would be a small element of randomness even in citation networks. The equivalent scenario of 'surfing' of World Wide Web in citation networks is a person (say, an academic) browsing papers of interest by following the citation links. The value $\alpha = 0.9$ signifies that after following ten links of citations, the academic would start her search from another random paper in the citation network $(1/(1 - \alpha) = 10)$. It is imaginable that even the most tenacious academic would lose interest after following citations through ten links, and would start her search/review from another paper in the literature (another 'random' point in the citation network). From a practical point of view, $\alpha < 1$ is necessary to simulate the pagerank process, as $\alpha = 1$ would result in all nodes having zero pagerank value at the beginning and hence all 'endorsements' will be zero. For these reasons, we have set a fairly high value for $\alpha$ but maintain it less than unity.

# References


[1] (2008). Quantum game theory google scholar page. URL http://scholar.google.com.au/citations?user=wkfPcaQAAAAJ&hl=en.
