# Supplementary material for: The Pagerank-Index: Going beyond Citation Counts in Quantifying Scientific Impact of Researchers
Source: PLoS One. 2015 Aug 19;10(8):e0134794. doi: 10.1371/journal.pone.0134794 (PMC4545754; doi:10.1371/journal.pone.0134794)
Supplement: S1 Supplementary Material — (PDF) [file pone.0134794.s002.pdf]

The pagerank-index: Going beyond citation counts in quantifying scientific impact of researchers
Supplementary material

Upul Senanayake, Mahendra Piraveenan, Albert Zomaya

May 3, 2015

This document provides some supplementary information, including additional results which could not be included in the paper due to space restrictions.

Citation and collaboration networks

The following tables present the topological properties for the citation and collaboration networks generated during the three scenarios of the simulation experiments. As the paper describes a typical experiment for each scenario, the properties of the network generated for that particular experiment are shown here. The properties are not averaged.

Property	Value
Number of nodes	25000
Number of edges	97555
Average degree	7.805
Characteristic path length	1.648
Clustering coefficient	0.0
Network diameter	8
Power law fit	$P(k) \sim k^{-1.847}$

Table 1: Characteristics of the citation network from scenario 1 in the simulation experiments.

Property	Value
Number of nodes	2134
Number of edges	86546
Network heterogeneity	0.844
Characteristic path length	2.287
Clustering coefficient	0.183
Network diameter	6
Network Density	0.038

Table 2: Characteristics of the collaboration network from scenario 1 in the simulation experiments.

Property	Value
Number of nodes	25000
Number of edges	20015
Average degree	1.601
Characteristic path length	1.0
Clustering coefficient	0.0
Network diameter	1
Power law fit	$P(k) \sim k^{-1.503}$

Table 3: Characteristics of the citation network for the second scenario in simulation experiments.

Property	Value
Number of nodes	2253
Number of edges	214126
Network heterogeneity	0.782
Characteristic path length	2.235
Clustering coefficient	0.278
Network diameter	6
Network Density	0.084

Table 4: Characteristics of the collaboration network for the second scenario in simulation experiments.

Property	Value
Number of nodes	25000
Number of edges	20431
Average degree	1.635
Characteristic path length	1.0
Clustering coefficient	0.0
Network diameter	1
Power law fit	$P(k) \sim k^{-0.947}$

Table 5: Characteristics of the citation network for the third scenario in a single run of simulation experiments.

Property	Value
Number of nodes	1662
Number of edges	78280
Network heterogeneity	0.966
Characteristic path length	2.246
Clustering coefficient	0.269
Network diameter	5
Network Density	0.057

Table 6: Characteristics of the collaboration network for the third scenario in a single run of simulation experiments.

Property	Value
Number of nodes	27770
Number of edges	352285
Average degree	25.372
Characteristic path length	6.460
Clustering coefficient	0.156
Network diameter	33
Power law fit	$P(k) \sim k^{-1.585}$

Table 7: Characteristics of the citation network generated from HEP-TH dataset.

Property	Value
Number of nodes	13524
Number of edges	24883
Network heterogeneity	1.119
Characteristic path length	7.481
Clustering coefficient	0.489
Network diameter	26
Network Density	0.009

Table 8: Characteristics of the collaboration network generated from HEP-TH dataset.

The following figures present the corresponding degree-distributions for citation and collaboration networks.

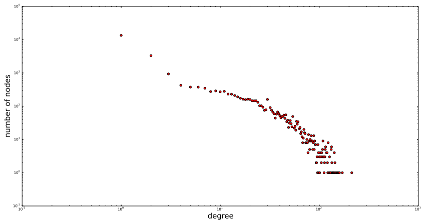

Figure 1: Degree distribution of the citation network for the first scenario in a single run of simulation experiments plotted in log-log scale.

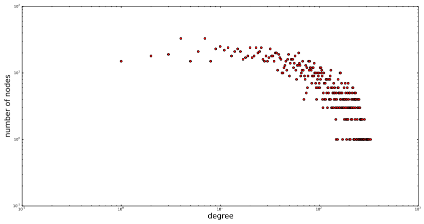

Figure 2: Degree distribution of the collaboration network for the first scenario in a single run of simulation experiments plotted in log-log scale.

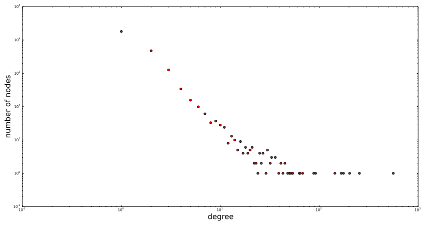

Figure 3: Degree distribution of the citation network for the second scenario in a single run of simulation experiments plotted in log-log scale.

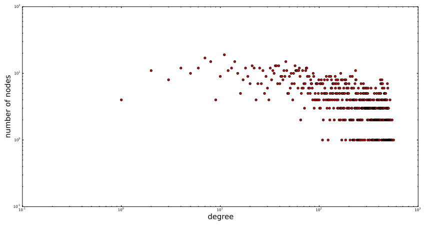

Figure 4: Degree distribution of the collaboration network for the second scenario in a single run of simulation experiments plotted in log-log scale.

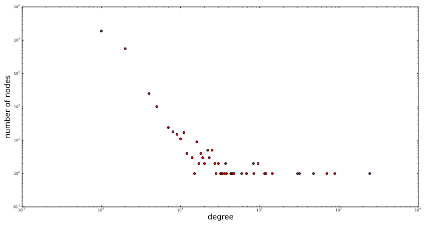

Figure 5: Degree distribution of the citation network for the third scenario in a single run of simulation experiments plotted in log-log scale.

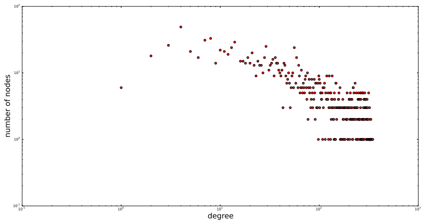

Figure 6: Degree distribution of the collaboration network for the third scenario in a single run of simulation experiments plotted in log-log scale.

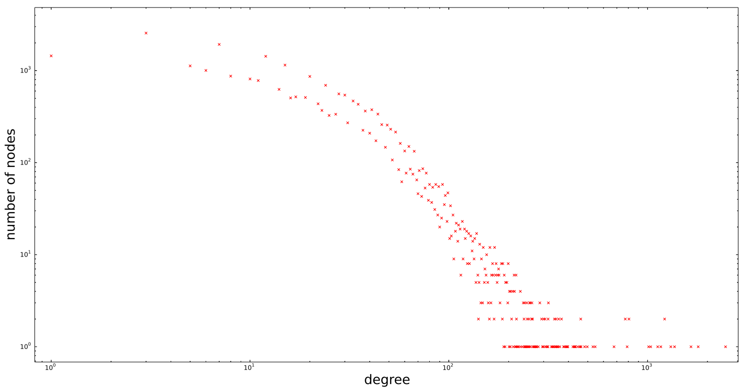

Figure 7: The degree distribution of the HEP-TH citation network plotted in log-log scale.
